# ParaMask: a new method to identify multicopy genomic regions, corrects major biases in whole-genome sequencing data

**DOI:** 10.1186/s13059-025-03836-8

**Published:** 2025-10-24

**Authors:** Bastiaan Tjeng, Male Arimond, Helene Bråten Grindeland, Andrea Dalla Libera, Andrea Fulgione

**Affiliations:** Max Planck Institute for Plant Breeding Research, Carl-von-Linne-Weg 10, 50829 Cologne, Germany

**Keywords:** Multicopy regions, Genomics, Repetitive sequences, Duplications, Transposable elements, Paralogs

## Abstract

Multicopy genomic regions are repeated sequences that can bias genomic analyses. Here, we present a method, ParaMask, to identify and filter multicopy regions in population-level genomic data of any species. The broad applicability of this method stems from a flexible Expectation-Maximization framework to detect excess heterozygosity while simultaneously fitting inbreeding levels. By combining this signature with read-ratio deviations, excess sequencing depth, and a clustering technique, our method attains high recall. We show that multicopy regions create biases that confound evolutionary genomic analyses and that by identifying these regions with our method and filtering them, we can correct these biases.

**Supplementary Information:**

The online version contains supplementary material available at 10.1186/s13059-025-03836-8.

## Background

Recent advances in DNA sequencing technology are revealing genomic variation across increasing numbers of species, populations, and individuals. However, some genomic regions still pose challenges to mapping and assembling sequenced fragments (reads) and to the unbiased identification of genotypes. Among these complex genomic regions, sequences that are repeated multiple times can be especially difficult [1]. These multicopy regions include tandem or segmental duplications, copy number variants, gene families with various paralog copies, transposable elements, and other repeats. Whether they are mapped to a reference genome with a single copy or assembled de novo, these regions tend to align to each other instead of mapping to their original genomic position, in which case the different copies are said to collapse [2]. The resulting mapping errors can bias the identification of small-scale genomic variants, like single-nucleotide polymorphisms (SNPs) [1, 3], with the possible exception of model organisms with well-characterized multicopy regions (including humans [4], Arabidopsis [5], and Drosophila [6]).

Multicopy regions can be prevalent in the genomes of some species. For instance, they are common in organisms with ancestral whole-genome duplication events (e.g., in plants [7, 8], vertebrates [9–11], insects [12], and yeast [13]), in species with abundant transposable elements (e.g., in some plants [14], insects [15], salamanders [16], and lungfish [17]), with segmental or tandem duplications (e.g., in amphioxus [18]), and/or other repeat elements. Multicopy regions can also have a pivotal role in adaptive evolution [19–21]. When genes are duplicated, one of the copies can maintain the ancestral function, while the other is free to evolve novel functions. For instance, the ancestral copy of the *ADH* gene in yeast converts acetaldehyde to ethanol, and a derived paralog performs the reverse reaction [21]. Furthermore, understanding gene copy-number variation can reveal genetic variants that contribute to adaptation, for instance, through their effect on gene product dosage [20, 21]. In *Arabidopsis halleri*, for instance, a triplication of the gene *HMA4* resulted in increased tolerance to heavy metals in the soil [22], and in *Arabidopsis thaliana*, copy-number variants at *NRAMP1* improved metal homeostasis [23]. Generally, the identification of multicopy regions is crucial for the correct and unbiased analysis of genomic data and also important to get a complete picture of the adaptive history of populations and species.

Multicopy regions can be identified from characteristic signatures in genomic data. When these regions are sequenced, especially with short reads, the number of reads mapping to the collapsed region (depth) will be in excess. This signature has been used to mask multicopy regions with thresholds based on the empirical distribution of genome-wide depth [3, 24–26]. However, depth distributions at single-copy and at multicopy regions largely overlap, resulting in high error rates for these approaches [27]. A second signature of multicopy regions is an excess of observed heterozygotes. When reads from multiple gene copies collapse during alignment, alleles that differ between the copies appear at intermediate alternative allele ratios (the proportion of reads associated with the alternative allele), resembling the signal typically observed in heterozygous genotypes. Different approaches test for an excess of heterozygotes compared to Hardy-Weinberg proportions using either exact tests [28] or thresholds for a negative inbreeding coefficient ($F_{IS}$) [27, 29]. These approaches have a high specificity, but also a low sensitivity, and their power decreases as the population frequency of SNPs deviates from 0.5. Multicopy regions can further be detected based on deviations in read ratios. When an individual is heterozygote on one copy and homozygote on the other, observed read ratios will be centered at 0.25 or 0.75. These deviations from expected read ratios at single-copy regions ($RR_{AA}=0.0$; $RR_{Aa}=0.5$; $RR_{aa}=1.0$) can improve the detection of multicopy regions. Especially for mid- to low-frequency SNPs, expected deviations are large, but so is the variance, which limits their detection at low frequencies [27]. While each of these methods can identify some multicopy regions, the best results are obtained by combining different signatures [3, 24, 27]. For instance, for genotype-by-sequencing (GBS) data, the HDplot method combines filtering based on excess heterozygosity and on read-ratio deviations [27]. This method was later extended in a stacks workflow [29] and in the package rCNV [30]. Another approach designed for low coverage data, ngsParalog, integrates a log-likelihood ratio test to detect excess heterozygosity [31]. Although these approaches are very valuable, we are still missing an established framework to identify multicopy regions in whole-genome data from any species. In particular, many species depart from random mating, for instance, due to selfing, assortative mating, population structure, and age structure [32]. In these species, using Hardy-Weinberg proportions to identify multicopy regions is overly conservative. In addition, the low prevalence of heterozygotes in inbred species decreases the power to test for read-ratio deviations.

Here, we developed ParaMask, an easy-to-use approach to identify multicopy regions in population-level whole-genome data. By modeling heterozygote frequencies in an Expectation-Maximization (EM) framework, we fit unknown levels of inbreeding to the data and avoid assumptions on random mating. This method combines information on excess heterozygosity, excess sequencing depth, read-ratio deviations, and clustering of multicopy SNPs. These signatures are easily accessible in a standard genomic *vcf* file, which is the only input required. We applied this approach to genomic data sets of four different species and validated the results with simulations and with long-reads sequencing. Finally, we show that multicopy regions bias genomic summary statistics and evolutionary inference and that filtering these regions with our method corrects most of the bias.

## Results

### ParaMask

**Fig. 1:**
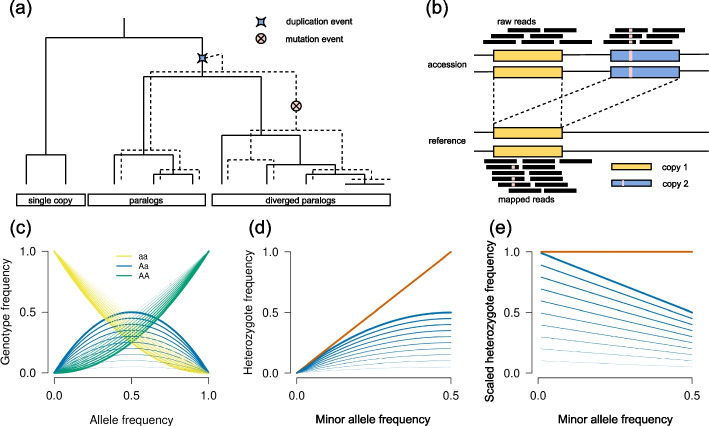
**a** Example of a genealogy with a duplication. The genealogy of the duplication (dashed line) is represented in the background of the genealogy of the ancestral copy (continuous line). The two genealogies may or may not differ, depending on linkage. **b** Example of sequencing and collapsing of multicopy regions. If the reference genome has a single copy (copy 1), and a sequenced accession has multiple copies (copies 1, 2), the copies can align to each other (collapse) resulting in increased depth and in an excess of observed heterozygotes. **c** Expected genotype frequencies with random mating (thick lines), and with increasing levels of inbreeding, $F_{IS}$ (thin lines represent $F_{IS}$ ranging between 0.1 and 0.9 with a step of 0.1; line thickness is proportional to $(1-F_{IS})$). Blue lines represent heterozygote frequencies, yellow and green lines homozygote frequencies. **d** Expected heterozygote frequencies as a function of the minor allele frequency (*maf*) at single-copy regions (blue lines as in panel **c**), and observed heterozygote frequencies at collapsed multicopy regions (red line). **e** Scaled heterozygote frequencies (*f*(*het*)/2*maf*). Lines are as in panel **d**

The method presented here, ParaMask, identifies genomic regions that are repeated multiple times, here called multicopy regions, in population-level, whole-genome data. When genomes are sequenced, the sequencing reads from the multiple copies map to each other (collapse). This results in characteristic signatures (summarized in Fig. 1), which are detected from a standard genomic data file (in.*vcf* format) in a three-steps approach. First, intermediate read ratios are observed when the different copies carry different alleles, which results in an excess of observed heterozygotes (Fig. 1b). In the first step, the method uses an Expectation-Maximization approach to identify single-copy and multicopy regions from heterozygosity levels, avoiding assumptions of random mating. This step has a high specificity, except for SNPs at low minor allele frequency (*maf*, the frequency of the rare allele), which have similar heterozygosity at single-copy and multicopy regions. Second, when one copy is heterozygote, and the other homozygote, read ratios deviate from expectations at single-copy regions. In the second step, we use this signature to refine the classification of SNPs at multicopy regions. Third, the collapse of multicopy regions results in excess sequencing depth (Fig. 1b). Finally, these signatures are not randomly distributed across the genome, but clustered in multicopy haplotypes. In the final step of ParaMask, we integrate the signatures of excess heterozygosity and depth, read-ratio deviations, and proximity among multicopy SNPs, to identify breakpoints between multicopy and single-copy regions. The three steps of the method are explained in the “Methods” section and in Additional file 1: Supplementary Text.

### ParaMask correctly classifies a high proportion of SNPs in simulations with single-copy regions and duplications

**Fig. 2:**
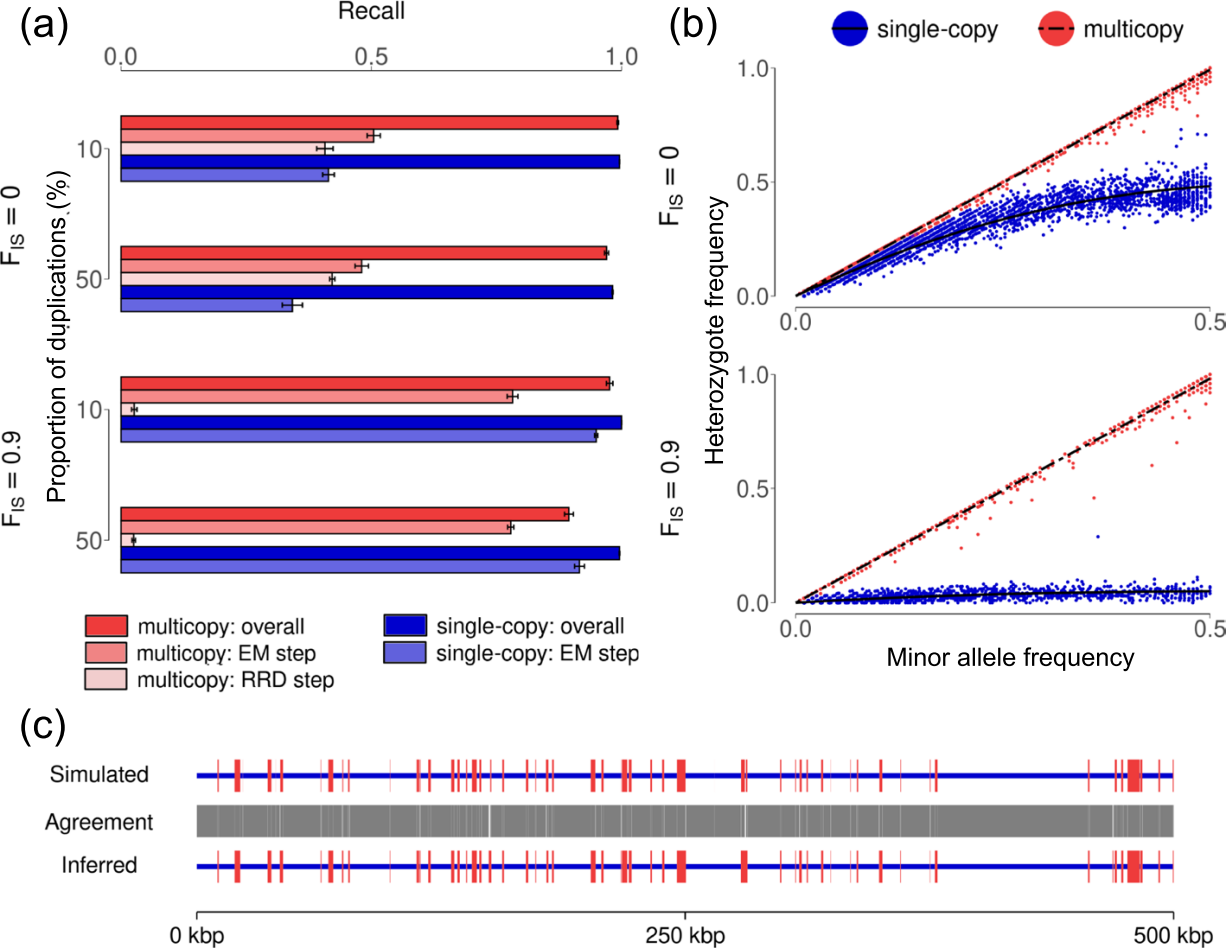
**a** Recall for single-copy (blue) and multicopy SNPs (red) in simulations with 10% or 50% duplications, and random mating or inbreeding ($F_{IS} = 0.0; 0.9$). The bars represent recall for the overall ParaMask procedure (dark colors), after only the first EM step (mid-to-light colors) and after testing for read-ratio deviations (RRD, only for multicopy SNPs, light colors). **b** Heterozygote frequency as a function of the minor allele frequency, colored in a gradient scale proportional to the posterior distribution of the latent factor (weights) at the final EM iteration. The color scale varies from red (SNPs likely to belong to multicopy regions) to blue (SNPs likely to belong to single-copy regions). Simulations with 10% duplications and random mating are shown at the top, and with inbreeding at the bottom. Curves represent the model fit for the relationship between heterozygote and allele frequencies at single-copy (solid line) and multicopy (dashed line) SNPs. **c** Example of a simulated haplotype (top row, 500 kbp long) with duplications (red) interspersed among single-copy regions (blue), and the states inferred with ParaMask (bottom row). The center row (agreement) shows when the simulated and inferred states are the same (dark gray), and when they are not (white)

We tested the performance of ParaMask on simulated genomes with a mosaic of duplicated haplotypes representing multicopy regions (10% of the sequence length) interspersed among single-copy regions, with random mating or inbreeding ($F_{IS} = 0.0; 0.9$), and in three replicates (details in the “Methods” section). In simulations with random mating, the full ParaMask procedure had a total recall (defined as the fraction of SNPs that were correctly classified) of 99.5% on average across replicates (range: 99.4% to 99.6%, over 7067 SNPs on average; Table 1). In single-copy regions, recall was 99.6% (range: 99.5% to 99.6%, over 5274 SNPs), and in duplicated regions 99.2% (range: 99.1% to 99.4%, over an average of 1793 SNPs on 97 to 128 duplicated haplotypes across replicates; Fig. 2a). To understand the relative contribution of the different signatures used in ParaMask, we analyzed the results separately for the three steps. Respectively for single-copy regions and duplications, the first EM-based step classified on average 41.4% and 50.5% of the SNPs correctly, 0.5% and 0.3% incorrectly, and 58.1% and 49.3% remained uncertain and were mostly at low frequency (Additional file 2: Fig. S1). The EM iterations, convergence and log-likelihood based classification are illustrated in Fig. 2b and Additional file 2: Fig. S1. In the second step, which tests for read-ratio deviations, on average an additional 40.7% of the SNPs in duplicated regions were correctly classified (range: 39.7−42.6%). The final step of clustering multicopy haplotypes accounted for the remaining proportion up to the total recall of 99.5%. An example of a simulated sequence, and the states inferred with ParaMask is given in Fig. 2c. In simulations with inbreeding, ParaMask had a total recall of 99.4% (range: 99.2% to 99.5%, over 3226 SNPs; Table 1). In single-copy regions, recall was 100% in each replicate (over 2367 SNPs), and in duplicated regions 97.6% (range: 96.9% to 98.2%, over 859 SNPs on 76 to 123 duplicated haplotypes across replicates; Fig. 2a). The EM-based step correctly classified 94.9% and 78.2% of the SNPs respectively in single-copy regions and duplications, 0.1% and 0.4% incorrectly, and 5.0% and 21.4% remained uncertain (Fig. 2a, Additional file 2: Fig. S2). The proportion of SNPs classified correctly at this step was 2.1-fold higher (single-copy: 2.3-fold; multicopy: 1.6-fold) than in simulations with random mating. The test for read-ratio deviations correctly classified an additional 2.6% of SNPs in duplications (range: 2.1−3.1%), a 16-fold lower proportion than with random mating (Fig. 2a).

**Table 1:** Average proportion of SNPs across replicates inferred as single-copy or multicopy (inferred category) in simulated single-copy or duplicated regions (simulated category) for different proportions of duplications (10% and 50%) and inbreeding coefficients ($F_{IS} = 0.0; 0.9$)

		*Simulated category*				Recall
		Single-copy SNPs		Multicopy SNPs		
*Parameters*		*Inferred category*				
% duplicated	*F*_*IS*_	Single-copy	Multicopy	Single-copy	Multicopy	All
10%	0.0	99.58%	0.42%	0.78%	99.22%	99.49%
10%	0.9	100%	0.00%	2.40%	97.60%	99.36%
50%	0.0	98.19%	1.81%	3.02%	96.98%	97.28%
50%	0.9	99.58%	0.42%	10.53%	89.47%	91.94%

We further tested the limits of ParaMask by simulating genomes with a very large proportion of duplications, 50% of the sequence length after collapsing, which corresponds to 66.7% before collapsing. In simulations with 50% duplications and random mating, average total recall was 97.3%, which is 3% lower than with 10% duplications (range across replicates: 96.9% to 97.5%, over 11708 SNPs on average; Table 1). In single-copy regions, recall was 98.2% (range: 98.1% to 98.3%, over 2887 SNPs), and in duplicated regions 97.0% (range: 96.5% to 97.6%, over 8821 SNPs on 488 to 526 duplications across replicates; Fig. 2a). In simulations with 50% duplications and inbreeding, total recall was 91.9%, which is 7.4% lower than with 10% duplications (range: 91.32% to 92.5%, over 5519 SNPs; Table 1). Recall was 99.6% in single-copy regions (range: 99.5% to 99.6%, over 1349 SNPs), and 89.5% in duplicated regions (range: 88.5% to 90.3%, over 4170 SNPs on 470 to 540 duplications across replicates; Fig. 2a). The relative contribution of the three steps of ParaMask are shown in Fig. 2a.

Finally, we assessed the performance of ParaMask with different sample sizes (10, 15, 20, 50, and 100 diploid individuals), and average sequencing depth (3×, 5×, 8×, 12×, and 20×) using simulations with 10% duplications, and with random mating and inbreeding. With a minimum sample size of 15 and 5× depth, total recall was 93.8% with random mating and 97.8% with inbreeding (Additional file 2: Fig. S3). With a sample size of 10 and random mating, the EM failed to converge, and with inbreeding the total recall was 87.0% with 10 samples and 5× depth, and 94.5% with 15 samples and 3× depth. For single-copy regions, the recall was high across all parameters explored (minimum average recall of 99.0%). At multicopy regions, recall decreased with smaller sample sizes and with lower depth (with random mating, 15 samples and 5× depth recall was 78.4%; with inbreeding, 15 samples and 3× depth recall was 86.6%; Additional file 2: Fig. S3 and Additional file 3: Table S1).

Overall, the EM-based step of ParaMask contributed more to correct SNP classification in simulations with inbreeding, while the test for read-ratio deviations contributed more to simulations with random mating (Fig. 2a). Therefore, these signatures are complementary to each other, and they result in high recall across the parameter space.

### ParaMask identifies multicopy regions in genomic data from diverse species

**Fig. 3:**
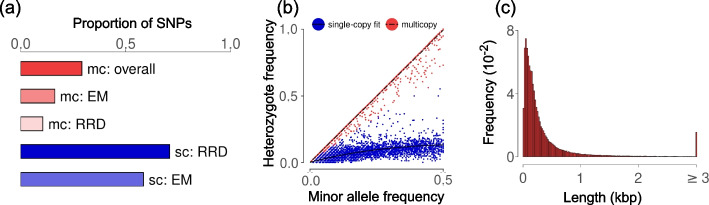
Identification of single-copy and multicopy regions in *A. alpina* populations with ParaMask. **a** Proportion of SNPs identified as multicopy (mc) or single-copy (sc). The bars represent proportions for the overall ParaMask procedure (dark colors), after only the first EM step (mid-to-light colors) and after testing for read-ratio deviation (RRD, only for multicopy SNPs, light color). **b** Heterozygote frequency as a function of the minor allele frequency, colored in a gradient scale proportional to the posterior distribution of the latent factor (weights) at the final EM iteration. The color scale varies from red (SNPs likely to belong to multicopy regions) to blue (SNPs likely to belong to single-copy regions). Curves represent the model fit for the relationship between heterozygote and allele frequencies at single-copy (solid line) and multicopy (dashed line) SNPs. **c** Histogram of the length distribution of multicopy haplotypes

To show that ParaMask can identify multicopy regions in different species, we tested it on a novel data set from the plant *Arabis alpina*, and on published data sets of *Oncorhynchus gorbuscha* (pink salmon) [33] and *Leptidea sinapis* (wood white butterfly) [34]. For *A. alpina*, we sequenced the whole genomes of 85 individual plants originally collected at two sites in Spain (ES03 and ES04, Additional file 3: Table S2). The average genome-wide sequencing depth was 20.4× (range: 18.2×–25.7×). After filtering for genotype quality ($\ge 30$ per individual and site), minimum depth ($\ge 5$ per individual and site), and missing values ($\le 0.1$ per site across individuals), we retained 2,972,173 biallelic SNPs. By running ParaMask on this data set, we identified 102,303 multicopy regions, for a total sequence length of 45.5 Mbp (14.6% of the reference sequence). These multicopy regions accounted for 29.1% (864,562) of all analyzed SNPs, of which 55.9% were identified in the first EM-based step, 36.3% after testing for read-ratio deviations, and 7.8% after clustering haplotypes (Fig. 3a). The posterior distribution of weights after convergence of the EM is shown in Fig. 3b. The distribution of read-ratio deviations is shown in Additional file 2: Fig. S4. Within ParaMask, we estimated a threshold for distances among SNPs within haplotypes at 254 bp in this data set, which is used in the clustering step. The distribution of haplotype lengths aligned approximately to an exponential decay with average length 445 bp (Fig. 3c), suggesting that many multicopy regions stem from repetitive regions.

For *O. gorbuscha* and *L. sinapis*, details on data handling and SNP filtering can be found in Additional file 1: Supplementary Text. For *O. gorbuscha*, we used 69 individuals and 987,697 biallelic SNPs after filtering. ParaMask identified 13,743 multicopy regions, with a total sequence length of 119.3 Mbp (5.5% of all autosomes). Multicopy SNPs accounted for 20.8% (205,642) of all SNPs, of which 29.3% were identified in the EM-based step, 58.1% in the test for read-ratio deviations, and 12.6% after clustering haplotypes (Additional file 2: Fig. S5a). The posterior distribution of weights after convergence of the EM is shown in Additional file 2: Fig. S5b. The threshold for distances among SNPs within multicopy haplotypes was estimated to be 596 bp. Multicopy regions were on average 8.7 kbp long, and the distribution of haplotype lengths had two modes at 308 bp and at 570 bp (Additional file 2: Fig. S5c), after which it decayed approximately exponentially. Salmonid fishes share a relatively recent ancestral whole-genome duplication (around 80 million years ago) [35]. The subsequent partial rediploidization [36, 37] left traces of tetrasomy, mostly at telomeric regions [38, 39]. Consistent with this, multicopy regions identified by ParaMask in *O. gorbuscha* are abundant, relatively long, and they tend to localize on chromosome arms (e.g. NC_060177.1, NC_060188.1, Additional file 2: Fig. S6).

For *L. sinapis*, we used 78 individuals and 794,136 SNPs after filtering. The method identified 8855 multicopy regions, with a total sequence length of 17.5 Mbp (3.0% of the reference genome). Among all SNPs, 12.7% (101,076) were multicopy SNPs, and 26.6% of these were identified in the EM-based step, 69.2% int the test for read-ratio deviations, and 4.2% after clustering haplotypes (Additional file 2: Fig. S5d). The posterior distribution of weights after convergence of the EM is shown in Additional file 2: Fig. S5e. We estimated a threshold for distances among SNPs within haplotypes of 300 bp. The average length of multicopy haplotypes was 2.0 kbp and the length distribution had three modes at 32, 159 and 301 bp (Additional file 2: Fig. S5f).

Overall, our method can identify multicopy regions in genomic data sets of a variety of species and scenarios, including animals and plants, outcrossing (*O. gorbuscha* and *L. sinapis*) and selfing species (*A. alpina* [40]), species with abundant and rare multicopy regions (3% to 15% of the genome), as well as species with a recent ancestral whole-genome duplication (*O. gorbuscha*).

### Long-read sequencing of *A. alpina* validate multicopy regions identified with ParaMask

To validate the multicopy regions identified by ParaMask, we sequenced two of the *A. alpina* accessions used for short reads, ES03-014 and ES04-014, with PacBio Hifi long reads. Compared to the short reads used in ParaMask, long reads are more likely to span the breakpoints of structural variants (SV), including multicopy regions, thereby allowing for their identification. To identify SVs in the long reads, we first aligned the long reads to the reference genome and called structural variants, and second, we assembled the genomes de novo, and screened the assemblies for multicopy regions.

#### Structural variant calling based on long reads validates multicopy and single-copy regions identified with ParaMask

**Fig. 4:**
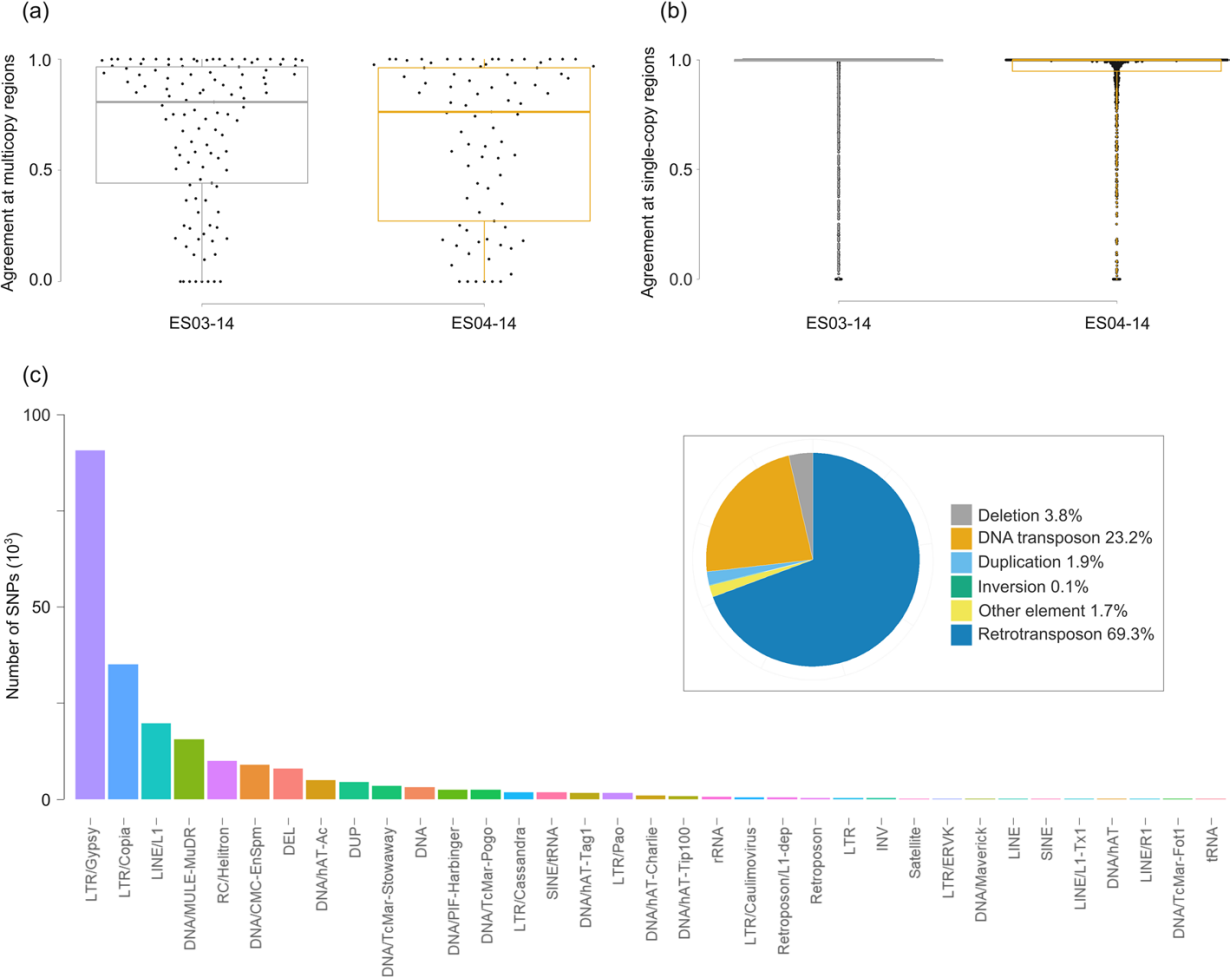
Comparison between ParaMask calls on genomic data of *A. alpina*, and structural variants (SV) calling and repeats calling from long reads in two accessions (ES03-014, ES04-014). **a** Proportion of SNPs within duplications identified by both sniffles2 and cuteSV in the long reads, that are also inferred to be in multicopy regions by ParaMask. Single points represent single duplications, and boxplots show median, 25th and 75th quantiles of the distribution. **b** Proportion of SNPs in genomic regions that do not overlap any SV, that are also inferred to be in single-copy regions by ParaMask. Single points represent single regions; and boxplots show median, 25th and 75th quantiles of the distribution. **c** Number of SNPs in multicopy regions called by ParaMask that overlap different structural variants and/or repeat classes identified with RepeatMasker on the long-reads assemblies (“Methods” section). The proportion of SNPs overlapping main classes are shown in the inset

For reference-based structural variant calling from long reads, we used two methods, cuteSV and sniffles2. In the accessions ES03-014 and ES04-014, cuteSV identified 548 and 394 duplications, 32,879 and 40,356 deletions, 30,642 and 36,487 insertions, and 149 and 141 inversions, respectively. Sniffles2 identified 305 and 219 duplications, 15,850 and 20,123 deletions, 13,303 and 17,795 insertions, and 221 and 271 inversions, respectively (Additional file 3: Table S3). Among these SVs, we focused on duplications, which are the only SV class identified here that always represents multicopy regions. Other SV classes may also tag multicopy regions (e.g., multicopy transpositions, or deletions when they represent excisions of multicopy transposons), but not always. We further merged the duplications that were identified by both methods, which increased our confidence in their presence (details in the “Methods” section). This resulted in a total of 117 and 83 high-confidence duplications with median length of 6785 bp (range: 110 bp to 84.0 kb) and 7459 bp (range: 380 bp to 49.7 kb), respectively in ES03-014 and ES04-014. Across these high-confidence duplications, the great majority was tagged by ParaMask with at least one multicopy SNP (in ES03-014: 89.7% of the duplications, or 100 out of 117; in ES04-014: 90.3%, or 75 out of 83). Within each duplication, ParaMask classified a median of 80.59% and 76.15% of the SNPs as multicopy, in ES03-014 and ES04-014, respectively (Fig. 4a, Table 2, Additional file 3: Table S4). We note that the remaining SNPs within SVs might have been classified as single-copy because they occurred at duplications that segregate at low population frequency, and/or due to the challenging identification of breakpoints between single-copy and multicopy regions. Furthermore, although we used two different methods, SV calling is prone to errors. Consistent with the low population frequency of SVs, the multicopy SNPs adjacent to single-copy SNPs within SVs were heterozygote in fewer individuals than genome-wide multicopy SNPs (a median of 1 heterozygote individual within SVs, and of 6 genome-wide, Additional file 1: Supplementary Text, and Additional file 2: Fig. S7).

**Table 2:** Median agreement between genomic regions with no identified structural variant and duplications called by both sniffles2 and cuteSV, and ParaMask calls for single-copy and multicopy SNPs

	*Structural variant calling category*			
	Single-copy region		Duplication	
	*ParaMask category*			
Accession	Single-copy SNPs	Multicopy SNPs	Single-copy SNPs	Multicopy SNPs
**ES03-014**	> 99.9%	< 0.01%	19.41%	80.59%
**ES04-014**	> 99.9%	< 0.01%	23.85%	76.15%

We also generated a set of high-confidence single-copy regions, where we expected that ParaMask would not identify any multicopy SNPs. For this, we focused on genomic regions where cuteSV and sniffles2 called no SV based on the long reads. Respectively in ES03-014 and ES04-014, we identified 2694 and 1231 high-confidence single-copy regions, with median length 3186 bp (range: 35 bp to 234.6 kb), and 5569 bp (range: 35 bp to 209.6 kb). ParaMask calls were, as expected, very rare in these regions, with a median $>99.9\%$ of the SNPs classified as single-copy in both genomes (Fig. 4b, Table 2, Additional file 3: Table S4).

#### De novo assembly of *A. alpina* genomes from long reads shows evidence of collapsed multicopy regions

As a complementary approach to reference-based SV calling, we generated de novo genome assemblies of ES03-014 and ES04-014, using a combination of Hifiasm and Flye (“Methods” section). The N50 length of the assembled contigs was 8.3 and 6.3 Mbp, and contigs were scaffolded to the reference genome to chromosome level. The final size of the genome assemblies was 335.6 Mbp and 356.9 Mbp, respectively, with a genome completeness of 97.9% and 98.4%, based on BUSCO (Additional file 3: Table S5). To identify collapsed multicopy regions, we mapped the raw Hifi reads to their corresponding assemblies, and we screened for genomic regions with excess depth compared to the genome-wide average of 36.7× and 56.7×, respectively. We detected regions of excess depth ranging from twice the genome-wide average, to peaks exceeding 1000× (Additional file 2: Figs. S8 and S9), which shows that multicopy regions also collapse in de novo assemblies with long reads. Among these regions, we found blast hits for genomic fragments of mitochondria and chloroplast, ribosomal DNA (rDNA) clusters, and copy-number variants including multicopy gene families.

#### Multicopy regions called by ParaMask include transposable elements, gene families of paralogs, repeats and structural variants

In order to understand the nature of the multicopy regions identified by ParaMask, we analyzed their overlap with genes that are annotated in the reference genome, with a repeat mask, and with reference-based SV calling from long reads, focusing on the accessions ES03-014 and ES04-014 (details in Additional file 1: Supplementary Text). The multicopy regions identified by ParaMask were significantly enriched for genes related to 22 gene ontology (GO) categories (based on topGO [41] Additional file 3: Table S6). These included genes related to the integration of DNA segments into chromosomes (DNA integration) which can be related to the activity of transposable elements (Additional file 1: Supplementary Text). Furthermore, we found an enrichment of defense response genes, including genes related to virus-induced gene silencing and genes related to defense response against bacteria. Among the multicopy regions identified by ParaMask and present in the accessions ES03-014 and ES04-014, 75.0% overlapped repeat elements and/or SVs in our merged set (Fig. 4c). Among these, 69.3% were retrotransposons, mostly long terminal repeat (LTR) *Gypsy* and *Copia* elements, and non-LTR *Line* elements, 23.2% were DNA transposons like *Mule* and *Helitrons*, 3.8% were deletions, which may include transposable elements excisions, 1.9% were duplications, and 1.7% were repeat elements such as those encoding transfer RNA (tRNA), ribosomal RNA (rRNA), and satellite repeats. The rest accounted for a small proportion of the regions ($< 0.1\%$; Fig. 4c).

### Comparison between ParaMask and other available methods

We compared the performance of ParaMask with the alternative methods rCNV [30] and ngsParalog [31, 42] on whole-genome data for *A. alpina*, and on simulated data with 10% duplications, and with random mating and inbreeding. Additionally, we evaluated its performance on a RAD-seq data set of Chinook salmon [43], for which a high-confidence set of multicopy regions is available [44], and compared the results to previous reports from rCNV and HDplot [27, 30].

In the analysis of empirical data for *A. alpina* with rCNV, 18.0% of the SNPs were classified as multicopy (535,042 SNPs out of 2,972,173), 30.7% as single-copy (911,317 SNPs), and 51.3% remained unclassified and therefore uncertain (1,525,814 SNPs; Additional file 3: Table S7). Among the SNPs that were classified in rCNV, 93.6% were classified in the same category by ParaMask (1,354,435 SNPs), including 34.0% of SNPs classified as multicopy (490,985 SNPs), and 59.7% of SNPs classified as single-copy (863,459 SNPs) by both methods (Additional file 3: Table S8). To validate the methods, we focused on the 117 and 83 high-confidence duplications identified with SV-calling from long reads (described above, and which had in total 17,361 and 12,237 overlapping SNPs). Because duplications identified from long reads can segregate at low population frequency, and because of possible errors in SV calling, we focused here on the relative comparison among methods in the proportion of SNPs classified as multicopy per duplication. Within duplications, rCNV classified a median of 36.8% and 42.9% of SNPs as multicopy, and 47.2% and 42.6% of the SNPs as uncertain (Additional file 2: Fig. S10). In comparison, ParaMask classified a median of 80.6% and 76.2% SNPs as multicopy (Fig. 4a). When focusing on the 48.7% of SNPs that were classified in rCNV, ParaMask and rCNV obtained similar results, with a median of 87.4% and 83.3% multicopy SNPs with rCNV, and 87.7% and 87.3% with ParaMask (Additional file 3: Table S9).

For ngsParalog, we repeated the analysis with Benjamini-Hochberg (BH) correction for multiple hypothesis testing with significance threshold $p<0.001$ (as in [42]), and with Bonferroni correction and $p<0.05$ (as in [31]). NgsParalog identified 34.8% of all genome-wide SNPs as belonging to multicopy regions with BH correction, and 26.7% with Bonferroni correction (respectively 1,033,268 and 2,971,902 SNPs out of 2,971,902), in comparison to 29.1% in ParaMask (864,562 SNPs out of 2,972,173; the total differs slightly due to internal SNP calling in ngsParalog; Additional file 3: Table S7). NgsParalog and ParaMask agreed in the classification of 82.4% and 84.5% of all SNPs with BH and Bonferroni correction respectively, including 23.1% and 20.1% of SNPs classified as multicopy (686,737 and 598,024 SNPs), and 59.3% and 64.4% of SNPs classified as single-copy (1,760,819 and 1,912,774 SNPs) by both methods (Additional file 3: Table S8). Within duplications, ngsParalog classified as multicopy a median of 58.3% and 52.1% of SNPs with BH correction, and a median of 46.9% and 38.1% with Bonferroni correction (Additional file 2: Fig. S11, Additional file 3: Table S9).

In the analyses on simulations with random mating, a sequencing depth of 10$\times$, and a sample size of 100 individuals, 31.7% of single-copy SNPs, and 27.3% of multicopy SNPs were unclassified with rCNV on average across replicates (over an average total of 7067 SNPs). The recall with rCNV was 69.2% for all SNPs (68.2% for single-copy SNPs and 72.0% for multicopy SNPs), and 99.6% for classified SNPs (Additional file 3: Table S10). On the same simulations, the recall of ngsParalog was 96.2% with BH correction (99.7% for single-copy SNPs, and 86.0% for multicopy SNPs), and 94.8% with Bonferroni correction (99.8% for single-copy SNPs, and 80.7% for multicopy SNPs; Additional file 3: Table S11). In comparison, as described above, the recall of ParaMask on the same simulations was 99.5% for all SNPs (99.6% for single-copy SNPs, and 99.2% for multicopy SNPs; Table 1). In simulations with inbreeding, on average 80.8% of single-copy SNPs and 29.3% of multicopy SNPs were unclassified with rCNV (over an average total of 3226 SNPs; Additional file 3: Table S10). The recall of rCNV was 9.1% for all SNPs (0.51% for single-copy SNPs, and 32.6% for multicopy SNPs), and 27.5% for classified SNPs. The recall of ngsParalog was 86.2% with BH correction (99.9% for single-copy SNPs, and 47.1% for multicopy SNPs), and 85.5% with Bonferroni correction (99.9% for single-copy SNPs, and 44.7% for multicopy SNPs; Additional file 3: Table S11). In comparison, total recall of ParaMask was 99.4% for all SNPs on the same simulations (> 99.9% for single-copy SNPs, and 97.6% for multicopy SNPs; Table 1). Finally, with 5× depth and sample size of 15, the recall of rCNV was 39.2% with random mating (over an average total of 4805 SNPs) and 5.3% with inbreeding (over an average total of 2047 SNPs, Additional file 3: Table S10). The recall of ngsParalog was 87.4% and 85.0% with random mating, and 79.4% and 78.5% with inbreeding, respectively with BH and with Bonferroni corrections (Additional file 3: Table S11). In comparison, the recall of ParaMask was 93.8% with random mating and 97.8% with inbreeding, using the same set of simulations (Additional file 3: Table S1).

In the analyses of RAD-seq data of Chinook salmon, we compared ParaMask calls with experimentally validated copy number annotations from [44], and with the performance reported for HDplot and rCNV [27, 30] on the same data set (Additional file 1: Supplementary Text). Assuming that previous copy number annotations are true, total recall of ParaMask was 97.3% (91.5% for multicopy SNPs and 98.3% for single-copy SNPs, Additional file 2: Fig. S12, and Additional file 3: Table S12). In comparison, HDplot reported recall rates of 95% and 97% for multicopy and single-copy SNPs, respectively [27], and rCNV reported an overall accuracy of 96% and a recall of 99.15% for single-copy SNPs [30]. Although ParaMask is designed for whole-genome sequencing data, it achieved comparable recall rates to alternative methods.

Overall, ParaMask had a high sensitivity in comparison to other approaches, in addition to broadening the spectrum of target species by accommodating unknown levels of inbreeding.

### Multicopy regions bias genomic summary statistics in simulated data and filtering these regions with ParaMask mitigates the bias

To characterize the effect of multicopy regions on genomic summary statistics, we simulated genomes with variable proportions of duplicated sequences (between 0% and 50%, step size 5%, three replicates each). We then computed commonly used summary statistics of genomic diversity such as Watterson’s and Tajima’s estimators of theta ($\theta _W$ and $\theta _\pi$), Tajima’s *D* ($D_{Taj}$), the inbreeding coefficient ($F_{IS}$), and the allele frequency spectrum (AFS). If ParaMask correctly identifies multicopy regions, we would expect their exclusion from simulated data to mostly correct biases in summary statistics. To test for this, we inferred multicopy regions with ParaMask on the simulated data, and also computed summary statistics on the single-copy subset of each simulation, and on the simulated data excluding multicopy regions identified by ParaMask.

**Fig. 5:**
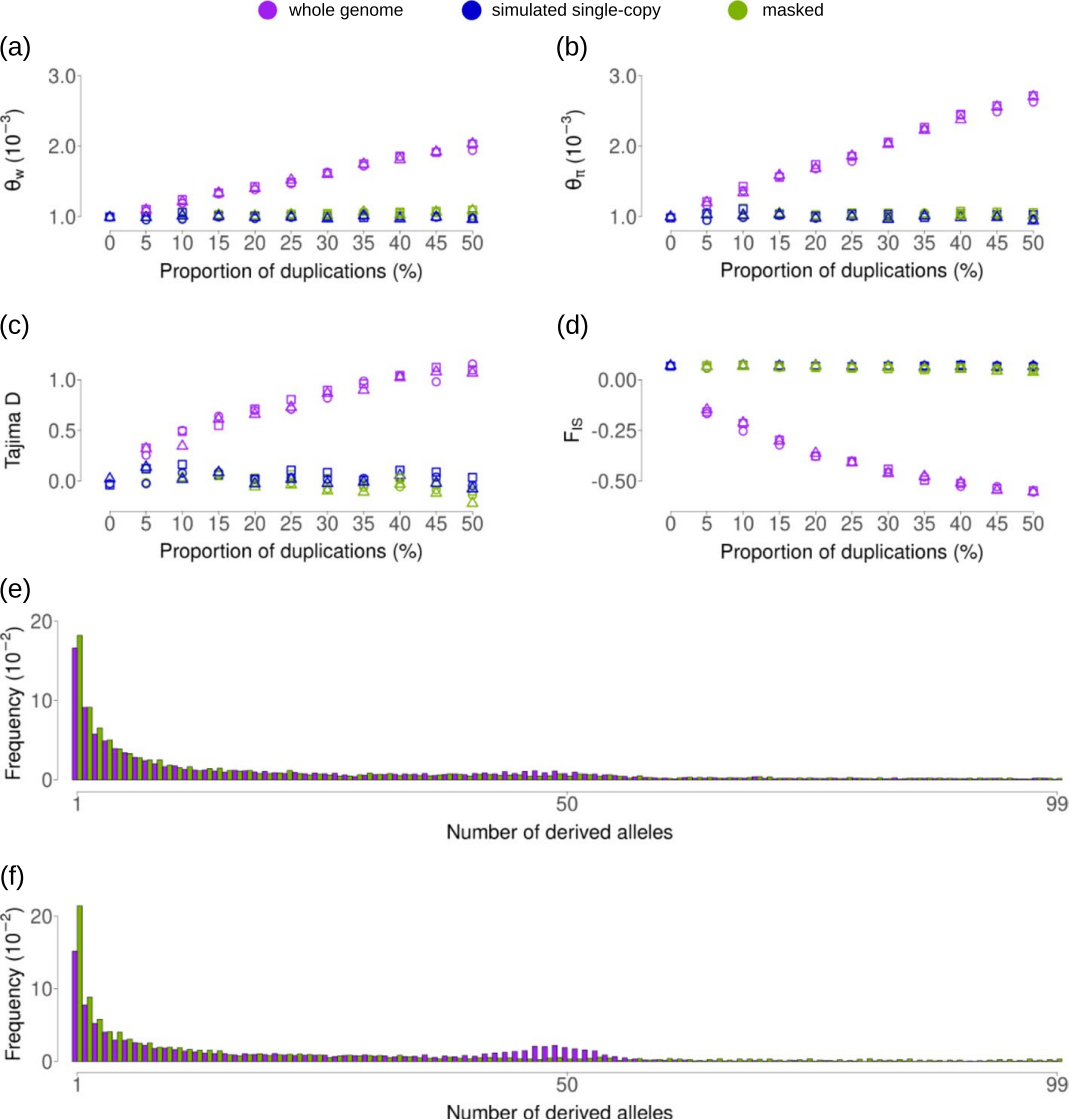
Effect of multicopy regions on genomic summary statistics in simulations. We simulated mosaic genomes with single-copy regions interspersed with 5% to 50% duplications (5% step), with random mating, in three replicates each (represented by symbols). We computed summary statistics on whole genomes (purple), on the single-copy subset of the genome (blue) and on the subset of the genome inferred to be single-copy with ParaMask (green). **a** Watterson’s estimator of theta, $\theta _{W}$. **b** Average pairwise differences, $\theta _{\pi }$. **c** Tajima’s D. **d** Inbreeding coefficient, $F_{IS}$. **e** Allele frequency spectrum (AFS) with 10% multicopy regions ($n=100$). Purple bars represent whole-genome AFS, green bars represent whole-genomes filtered for multicopy regions with ParaMask. **f** AFS with 50% multicopy regions ($n=100$). Colors as in panel **e**

In simulations with increasing proportions of duplications, $\theta _W$ was increasingly overestimated, and this bias increased approximately linearly. Compared to single-copy regions, estimates of $\theta _W$ ranged from 1.11-fold higher in simulations with 5% duplications to 2.04-fold higher with 50% duplications, on average across replicates (Fig. 5a). Because $\theta _W = S / a_k$, where *S* is the number of segregating sites, and $a_k$ is the total length of the coalescent tree given the number of genomes, *k* [45], this points to a higher number of segregating sites per base pair at multicopy regions. After filtering multicopy regions identified by ParaMask, most of this bias was corrected, but a slight bias of 0.41% (with 5% duplications) to 8.28% (with 50% duplications) remained. The average density of pairwise differences, $\theta _{\pi }$, was also increasingly overestimated with increasing proportions of duplications. Compared to single-copy regions, $\theta _{\pi }$ was 1.18-fold higher with 5% duplications, and 2.75-fold higher with 50% duplications (Fig. 5b). Filtering multicopy regions with ParaMask almost completely corrected this bias, leaving a remaining bias of 0.48% (5% duplications) up to 4.09% (10% duplications). The bias for $\theta _\pi$ was larger than the bias for $\theta _W$, implying an excess of intermediate-frequency SNPs at multicopy regions. Consistent with this, Tajima’s *D* ($D_{taj}= (\theta _\pi - \theta _W) / \sqrt{\sigma ^2}$ [46]) was also overestimated by 0.22 in simulations with 5% duplications compared to single-copy regions (corresponding to an increase of 2.5 standard deviations among replicates calculated on single-copy regions, $SD_{sc}$), and up to 1.14 with 50% duplications (or $+19.9$ $SD_{sc}$, Fig. 5c). After filtering with ParaMask, $D_{taj}$ was almost unbiased in simulations with 5% and 10% duplications ($<0.003$ difference, or $<0.04$ $SD_{sc}$). However, because of the slight remaining bias in $\theta _{W}$, $D_{taj}$ was slightly biased in simulations with 15% ($-0.01$ difference, or $-1.0$ $SD_{sc}$) to 50% duplications ($-0.12$ difference, or $-2.2$ $SD_{sc}$). The inbreeding coefficient $F_{IS}$ was increasingly underestimated with increasing proportions of duplications (Fig. 5d), implying an excess of heterozygosity at multicopy regions. Because $F_{IS}$ is bound between − 1 and 1, the relative increase in bias is larger with low proportions of duplications (with 5% duplications, $-0.22$ difference, or $-38.1$ $SD_{sc}$), and it levels out at higher proportions (with 50% duplications, $-0.62$ difference, or $-159.5$ $SD_{sc}$). After filtering with ParaMask, this bias was mostly corrected and the remaining difference ranged between − 0.0004 (or $-0.07$ $SD_{sc}$ with 5% duplications) and − 0.0179 (or $-4.6$ $SD_{sc}$ with 50% duplications). Finally, we assessed the allele frequency spectra (AFS) with 10% and 50% duplications, with and without filtering with ParaMask. In the AFS with multicopy regions, we detected an excess of intermediate-frequency variants, consistent with the larger bias in $\theta _{\pi }$ than in $\theta _{W}$ and with the overestimation of $D_{taj}$. These effects were more pronounced with 50% than with 10% duplications, and were mostly corrected after filtering with ParaMask (Fig. 5e, f). Overall, we have used simulations to show that multicopy regions bias genomic summary statistics, and that filtering them with ParaMask mitigates most of the bias.

### Multicopy regions bias genomic summary statistics in whole-genome data of *A. alpina*

We used whole-genome sequencing data of *A. alpina* to test whether multicopy regions result in similar biases as those detected in simulated data. For this, we computed the same set of summary statistics ($\theta _W$, $\theta _\pi$, $F_{IS}$, $D_{Taj}$, and the AFS) on the 85 *A. alpina* genomes sequenced with short reads, both using whole-genome data and after masking multicopy regions with ParaMask. In addition, we estimated nucleotide divergence ($F_{ST}$), and the joint allele frequency spectrum (jAFS) between the two populations (Methods section).

**Fig. 6:**
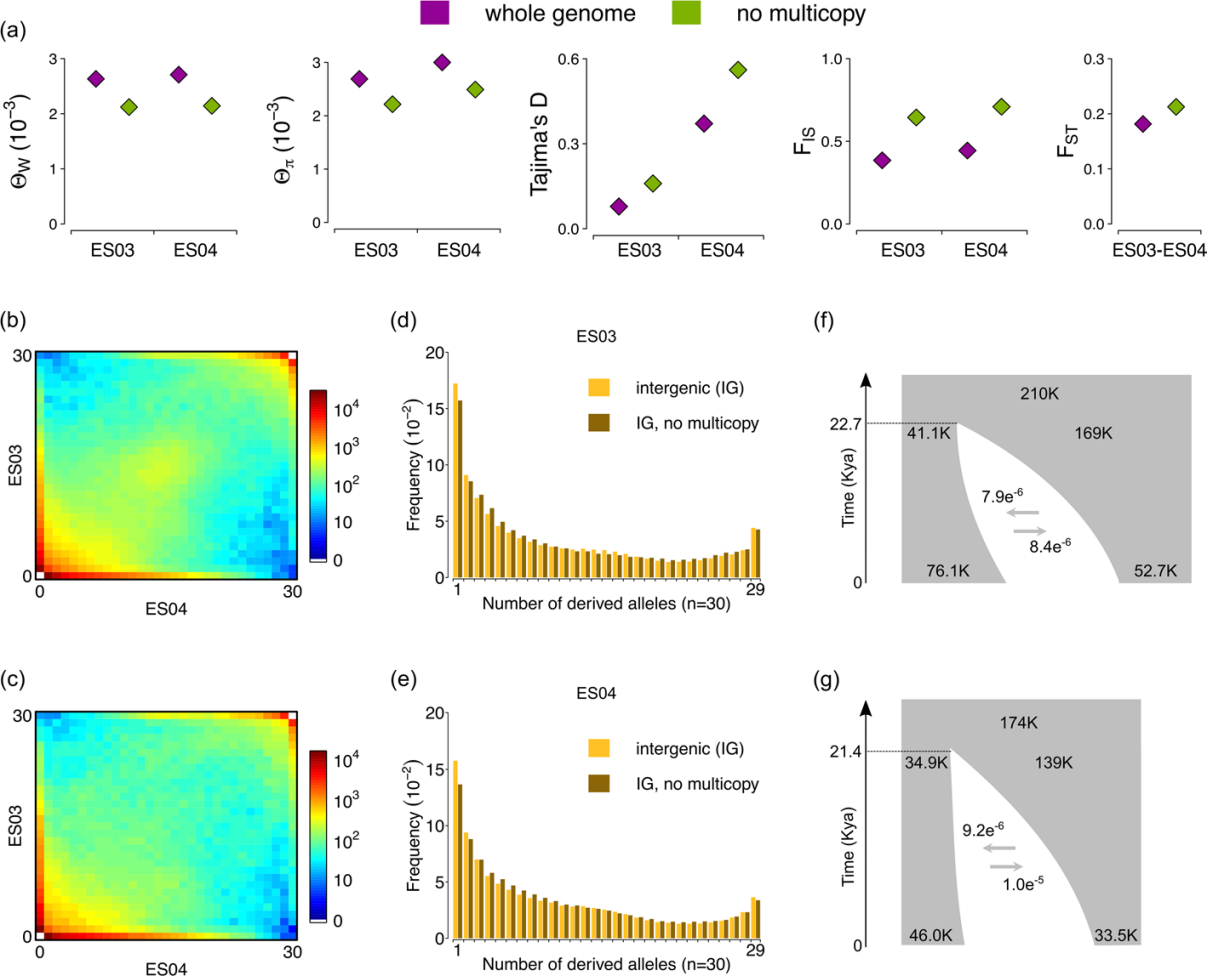
Effect of multicopy regions on summary statistics in 85 *A. alpina* genomes from two Spanish populations (ES03, ES04). **a** Summary statistics calculated on whole genomes (purple), and after filtering multicopy regions with ParaMask (green). **b** Intergenic joint allele frequency spectrum (jAFS) between populations ES03 and ES04. **c** Intergenic jAFS between populations ES03 and ES04 after filtering multicopy regions with ParaMask. **d** Allele frequency spectrum (AFS) for population ES03 on intergenic data (yellow), and after filtering multicopy regions with ParaMask (brown). **e** AFS for population ES04 on intergenic data (yellow), and after filtering multicopy regions with ParaMask (brown). **f** Schematic of the inferred demographic model and parameters optimised with *dadi*, based on intergenic jAFS, with no filter for multicopy regions. **g** Schematic of the inferred demographic model and optimised parameters, based on intergenic jAFS after filtering multicopy regions with ParaMask

Consistent with our results from simulations, multicopy regions in empirical data resulted in higher estimates of diversity ($\theta _{\pi }$ and $\theta _{W}$), and lower estimates of the inbreeding coefficient $F_{IS}$ (Fig. 6a). In the two *A. alpina* populations, estimates of $\theta _{\pi }$ and $\theta _{W}$ were respectively 1.21-fold and 1.25-fold higher with multicopy regions than after filtering with ParaMask. The magnitude of this overestimation was comparable to simulations with 10% duplications, where respectively $\theta _{\pi }$ and $\theta _{W}$ were on average 1.33-fold and 1.20-fold higher, similar to our estimate of 14.6% multicopy regions in the *A. alpina* populations. However, in empirical data, $\theta _{W}$ was more biased than $\theta _{\pi }$, which resulted in a 0.08 (ES03) and 0.19 (ES04) lower estimate of $D_{taj}$ without filtering multicopy regions. Estimates of $F_{IS}$ were 0.26 (ES03) and 0.27 (ES04) lower without filtering multicopy regions, consistent with an excess of heterozygosity (Fig. 6a). Additionally, we found that multicopy regions biased downward estimates of population differentiation, $F_{ST}$, which were 14.6% lower without filtering multicopy regions (Fig. 6a). Consistent with this, the proportion of SNPs that were polymorphic in both populations (shared polymorphisms) was higher with multicopy regions (43.9%) than after filtering with ParaMask (41.7%). This excess of shared polymorphisms was clustered at intermediate frequencies, around 15 out of 30 derived alleles in both populations, as evident in the jAFS (Fig. 6b, c). Shared polymorphisms at similar frequency are a hallmark of recent shared ancestry between populations, and therefore their excess at multicopy regions dilutes the signal of population differentiation. In the AFS within populations, multicopy regions had a slight excess of intermediate-frequency variants (around frequency 0.5) within populations, more evident for ES03 than for ES04 and consistent with simulations (Fig. 6d, e). In addition, and different from our simulations, we detected an excess of low-frequency variants (mainly singletons and doubletons), and a slight excess of nearly fixed variants (at 29/30 copies), likely due to mispolarization of singletons. These biases were largely corrected with ParaMask (Fig. 6d, e).

Allele frequency spectra (AFS and jAFS) are commonly used to infer demographic parameters, such as split times between populations, migration rates, and effective population sizes. Because multicopy regions create distortions in the AFS and jAFS, we tested whether they also bias AFS-based demographic inference. For this, we used the two jAFS of intergenic SNPs for the *A. alpina* populations, with and without exclusion of multicopy regions with ParaMask (Fig. 6b, c). For each jAFS, we tested four demographic models of increasing complexity, and for each model we fit parameters 200 times independently (details in “Methods” section) using dadi [47]. For both jAFS, the best supported model was an isolation-with-migration model with a split between populations followed by asymmetric migration and exponential changes in effective population size (Additional file 3: Tables S13, S14). However, the optimized model parameters differed. In the model optimized on data with multicopy regions, all five parameters for effective population size were inferred to be larger than after excluding multicopy regions with ParaMask (Fig. 6f, g, Additional file 3: Tables S13, S14). Split time and migration rates differed only marginally. This model converged to fit the excess of intermediate-frequency shared SNPs, evident in the unfolded and folded empirical jAFS, and in the model jAFS (Fig. 6f, Additional file 2: Fig. S13). Larger population sizes fit the increase in diversity, and they result in a reduced rate of drift. Therefore, they retain shared polymorphisms at intermediate frequencies for a longer time. Conversely, the model optimized after excluding multicopy regions converged to fit the lower proportion of intermediate-frequency shared SNPs (Fig. 6g, Additional file 2: Fig. S14). The residuals between model predictions and empirical data had a smaller range (− 25 to 10 versus − 40 to 10) and were more evenly distributed across allele frequencies after removing multicopy regions, resulting in a better fit (Additional file 2: Figs. S13, S14). Overall, consistent with the observed downward bias on $F_{ST}$, multicopy regions reduce the signal of population differentiation by increasing the proportion of shared polymorphisms at intermediate frequencies.

## Discussion

### ParaMask identifies multicopy regions in population-level, whole-genome sequencing data

Genomic regions that occur in multiple copies can align to each other (collapse) after sequencing, which results in errors in mapping and in variant calling. As a consequence, filtering multicopy regions is crucial for unbiased genomic analyses. Here, we present a method to identify and filter multicopy regions from population-level whole-genome sequencing data of potentially any diploid species. The broad applicability of this method is the first novelty introduced here, and it stems from a flexible Expectation-Maximization framework to detect excess heterozygosity while simultaneously fitting inbreeding levels. The second novelty is the integrative use of complementary signatures, which attains high power to detect multicopy regions across the parameter space.

The first signature we use to detect collapsed multicopy regions is an excess of heterozygosity. Here, we introduce an essential new feature that allows us to avoid the assumption of random mating, which is at the core of other methods that focus on this signature [27, 29, 30]. This is crucial for species with a selfing mating system, population structure, age structure, or any other source of inbreeding. We attain this flexibility by using an Expectation-Maximization framework to fit a mixture of two distributions of heterozygote frequencies as a function of minor allele frequencies, one due to single-copy and one to multicopy regions. This joint regression framework, in combination with a beta-binomial model, can fit unknown inbreeding levels as well as other idiosyncrasies of the data, for example due to complex population structure, and it can converge to random mating if the data supports this scenario. The use of a probabilistic SNP classification based on log-likelihood ratios instead of a binary classification in single-copy and multicopy SNPs provides a measure of statistical confidence, and avoids assigning uncertain SNPs based on arbitrary thresholds. Furthermore, our framework increases the power to detect mid- to low-frequency SNPs, although very rare SNPs remain challenging. We note that our approach differs from methods that filter SNPs [27, 29] or samples [30] on the basis of $F_{IS}$, which assume random mating and do not account for inbreeding. In simulations with inbreeding, our EM-based approach correctly classifies a 2.1-fold higher proportion of SNPs than with random mating. This is likely because inbreeding increases the difference between heterozygote frequencies at single-copy and at multicopy regions across allele frequencies (Fig. 2b). The addition of overdispersion in the model can account for increased variance due to sequencing errors, binomial sampling at heterozygote sites, noise in estimating allele frequencies due to low depth or low coverage, mapping bias and interlocus gene conversion (IGC) at multicopy regions. These factors are difficult to model explicitly, because the magnitude of their effects depends on parameters that are typically unknown (e.g., the rate of IGC), but they are easily accommodated by overdispersion.

The second signature we use is deviations in read ratios. This signature has been shown to improve the detection of multicopy regions in reduced representation sequencing data on outcrossing species [27, 30]. However, this signature relies on the presence of heterozygote genotypes, and these can be rare in inbred populations. Consistent with this, the contribution of read-ratio deviations to SNP classification in ParaMask was larger in simulations with random mating (40.7% contribution) than with inbreeding (2.6% contribution). Excess heterozygosity and read-ratio deviations are therefore complementary signatures across inbreeding levels, as well as across allele frequencies as described before [27]. Finally, our procedure of elongating multicopy haplotypes based on clustering and on excess sequencing depth introduces the possibility to identify multicopy SNPs at very low population frequencies, which is another advantage of our method compared to other approaches.

This approach has high recall with contiguous whole-genome sequencing data for at least 15 individuals, and with an average read depth of at least five. The recall drops for lower sample sizes, for lower read depth, and for fragmented sequencing, such as RAD-seq. Our approach can detect multicopy regions in two or more copies; however, the range of expected read ratios increases with the number of copies. As read ratios deviate from 0.5, some individuals may not be identified as heterozygote, and a simple approach based on excess coverage may be the most successful. Overall, by integrating complementary signatures, our method attains a high recall across allele frequencies, and in randomly mating and inbred species.

### Multicopy regions bias population genetics summary statistics and evolutionary inference

We have shown that multicopy regions bias estimates of genetic diversity ($\theta _\pi$ and $\theta _W$) upward, and estimates of the inbreeding coefficient ($F_{IS}$) and population differentiation ($F_{ST}$) downward. For Tajima’s *D*, we have detected an upward bias in simulated data, and a slight downward bias in empirical data. This difference likely stems from the expected downward bias in Tajima’s *D* in recent duplications due to a skew in the observed allele frequency spectrum, which evolves into an upward bias as mutations fix on one duplicated copy and become collapsed substitutions [48]. In our results, the collapsed substitutions are evident in the simulated data as peaks at intermediate frequencies in the allele frequency spectra (Fig. 5e, f), consistent with the ancient origin of these simulated duplications. In empirical data, these variants are also evident but less prominent (Fig. 6b–e), and we additionally detected an excess of rare variants and a negative Tajima’s D (Fig. 6a, d, e). This suggests that in *A. alpina*, multicopy regions of recent origin prevail, consistent with previous results that detected recent bursts of transposable elements [49]. IGC interferes with the accumulation of collapsed substitutions; thus, both $\theta _{\pi }$ and Tajima’s *D* are less biased with higher rates of IGC [2, 48]. However, this effect is limited to the so-called time of concerted evolution, where the loss of divergence due to IGC balances the increase in divergence due to mutations [50].

When multicopy regions are shared between populations, collapsed substitutions will be observed at intermediate frequency in each population, and their frequencies will not diverge over time due to drift. This effectively dilutes the signature of genomic divergence, leading to an underestimation of population differentiation (e.g., $F_{ST}$). Demographic inference methods may converge to different combinations of parameters that reduce estimated drift between populations, for instance underestimating split times, and/or overestimating population sizes and migration rates, consistent with our results on empirical data (Fig. 6f, g). As the time from the split increases, this scenario forms a genomic mosaic of single-copy regions that are richer in private variation, and multicopy regions that are richer in shared polymorphisms between populations or species. With increasing divergence times, collapsed substitutions will remain as trans-specific, shared polymorphisms, which may lead to wrong interpretations in evolutionary inference. If multicopy regions are prevalent, this scenario may resemble local barriers to gene flow (single-copy regions) on a background with higher migration rates (multicopy regions). This pattern has been associated with the idea of genomic islands of speciation or of divergence [51]. Conversely, when a genome is mostly single-copy with some interspersed multicopy regions, these may be wrongly inferred to be loci under long-term balancing selection. Balancing selection results in a series of signatures: (1) high sequence diversity; hence high estimates of $\theta$; (2) excess intermediate-frequency variants, and therefore high Tajima’s *D*; (3) reduced population differentiation ($F_{ST}$); (4) excess of trans-specific polymorphisms [52, 53]. These expected signatures are found at collapsed multicopy regions, which can therefore challenge the detection of long-term balancing selection. Curiously, some genes that are known to evolve under balancing selection are hotspots of rearrangements and form multicopy gene families. Some examples are genes involved in pathogen recognition at the major histocompatibility complex (MHC) in vertebrates [54, 55], in resistance to pathogens (R-genes) in plants [56], and in heterokaryon incompatibility in fungi [57]. While balancing selection at these loci is well established, a method like ours can help disentangle the effects of multicopy regions and of selection genome-wide. Overall, multicopy regions create a mosaic pattern of artifacts in genomic summary statistics. Therefore, masking these regions with a method like ParaMask is crucial for the unbiased inference of evolutionary histories and of selection.

### Multicopy regions identified by ParaMask include transposable elements, structural variants, gene families of paralogs and other repeat elements

We validated ParaMask calls on genomic data for *A. alpina* using long-reads sequencing. Among the multicopy regions identified by ParaMask, the majority were transposable elements, mostly LTR retrotransposons like *Gypsy* and *Copia* elements, and also *LINE* elements and DNA transposons like *MULE* and *Helitrons*. The classes of transposable elements identified here, and their abundances, are consistent with the ones previously identified in the *A. alpina* reference genome [49]. ParaMask calls also overlapped structural variants of different kinds, revealed with long-reads sequencing. Duplications are one kind of multicopy regions, but we also found overlap with deletions, and with a small proportion of inversions. Although different kinds of structural variants may create mapping errors and biases in SNP calling, some of the deletions identified here may be excisions of multicopy transposable elements. We also found ParaMask calls overlapping members of multicopy gene families, such as ribosomal DNA (rDNA) clusters, defense response genes, genes encoding transfer RNA (tRNA), and gene families with several paralogs in *A. alpina* such as *MADS AFFECTING FLOWERING (MAF)* and *MAF-RELATED (MAR)* gene clusters [58]. Finally, multicopy regions also overlapped genomic fragments of mitochondria and chloroplast, and satellite repeats. Analyses similar to these can be performed by the users downstream of ParaMask to identify the nature of multicopy regions in other species. Our results revealed a complex genomic ecosystem of multicopy regions, roughly consistent with a recent assessment across four fish species [42], although we expect the actual spectrum of variants to vary across different species. Our results also show that even in de novo assemblies based on long reads, multicopy regions can still collapse. We expect collapsed regions to be fewer than in short reads, and on average longer, because regions that are shorter than read length are likely to be correctly assembled.

## Conclusions

In the rapidly advancing field of empirical genomics, multicopy regions will remain a challenge. Here, we have devised an easy-to-use method for the identification of multicopy regions in population-level genomic data sets of any species. We have also identified the biases that multicopy regions introduce in genomic and evolutionary analyses, and we have shown that filtering these regions with our method corrects most of the bias. Furthermore, this method can be used to study the multicopy regions themselves, for instance gene families of paralogs or bursts of transposable elements. Whether it is used to filter multicopy regions from genomic data, or to focus on their contribution to adaptation, we suspect that a method like ours will assist the field of genomics for several years to come.

## Methods

### An Expectation-Maximization approach to identify single-copy and multicopy regions from heterozygosity levels

In the first step of ParaMask, we use heterozygosity levels to identify SNPs in single-copy regions, SNPs in multicopy regions, and SNPs that cannot be classified based on heterozygosity (uncertain). A novelty of our approach is that we avoid assumptions of random mating, which is crucial for species with unknown inbreeding levels. With random mating, genotype frequencies are expected to align to Hardy-Weinberg proportions at single-copy regions (thick lines in Fig. 1c), including heterozygotes ($E[f(Aa)] = 2p(1-p)$, where *p* is the allele frequency). With inbreeding, expected genotype frequencies depend on the inbreeding coefficient $F_{IS}$, and for instance, heterozygote frequencies decrease ($E[f(Aa)] = 2p(1-p)(1-F_{IS})$; thin lines in Fig. 1c represent increasing inbreeding levels). These same relationships hold for the minor allele frequency (*maf*), which varies between 0 and 0.5 (blue lines in Fig. 1d). At multicopy regions, the sites with different alleles on each copy are observed at intermediate read ratios, which are generally identified as heterozygote in SNP calling. As a consequence, and without IGC, every copy of the minor allele is observed in a heterozygote genotype, and $O[f(Aa)] = 2 maf$ (where *maf* is the observed minor allele frequency; red line in Fig. 1d). This prediction holds for multicopy regions in two or more copies. However, as the number of copies increases, the range of expected read ratios also increases, and some individuals with extreme read ratios may not be identified as heterozygote. For statistical purposes, it is beneficial to deal with outcome variables that depend linearly on the predictor variables, and that are bound to values independent from the predictor variables. We obtain this by dividing the heterozygote frequency by twice the *maf*, which is also the maximum possible heterozygote frequency given the minor allele frequency. We therefore use *f*(*Aa*)/2*maf* as outcome variable (Fig. 1e). With this transformation, all expected relationships are linear, and the upper bound of the outcome variable is one. Also, heterozygote frequencies at single-copy regions have an intercept and a slope of $(1-F_{IS})$ that can converge to one, when mating is random (Fig. 1e).

Here, we use these different relationships between heterozygote frequencies and allele frequencies to distinguish between single-copy and multicopy regions, with no assumptions on random mating. To do so, we simultaneously fit SNPs at single-copy and at multicopy regions genome-wide in a joint regression of heterozygote frequencies as a function of the minor allele frequency (*maf*), transformed as in Fig. 1e. For this regression, we use a beta-binomial model, parameterized by a mean and an overdispersion term, using a vectorized linear model (vglm) from the VGAM R package *v*1.1.8 [59]. The regression consists of first duplicating the data set, and assigning one replicate to fit SNPs at multicopy regions (level *D* of factor *Z*), and the other to fit SNPs at single-copy regions (level *K* of factor *Z*). Every SNP ($x_{i}$) is associated with a weight ($W_{x_{i}}$) between [0; 1], used as soft assignment to single-copy, or to multicopy regions. The initialization of weights is described in Additional file 1: Supplementary Text. For the mean we use the following model:

$$\begin{aligned} f(Aa) \sim Z + I((1-maf) * (Z==K)) \end{aligned}$$

The model fits an intercept for each level of factor *Z* and an interaction term *I* of level *K* (corresponding to single-copy SNPs) with $(1-maf)$. Because $(1-F_{IS})$ is independent of the *maf*, it is absorbed in the regression parameters for SNPs at single-copy regions, allowing the model to fit the inbreeding coefficient to the data (including when mating is random). For multicopy regions, the model fits a constant hazard ratio for *f*(*Aa*)/2*maf*, corresponding to a straight line (red line in Fig. 1e). Fitting overdispersion is discussed in Additional file 1: Supplementary Text. After fitting, we calculate probability densities from predicted means for SNPs at single-copy and at multicopy regions with the dbetabinom() function. Then we recalibrate weights and iterate these steps until likelihood convergence is reached, or for a user-specified number of iterations (details in Additional file 1: Supplementary Text). The probability densities from the last iteration are a measure of the likelihood of the model parameter *Z* given the observed heterozygous frequencies at the focal SNP and genome-wide. We use these probabilities to obtain the log-likelihood ratio as a measure of the relative support for a model where the SNP originated at a single-copy or at a multicopy region. The thresholds used for SNP classification are discussed in Additional file 1: Supplementary Text.

### Read-ratio deviations improve the identification of multicopy regions

In the second step of ParaMask, we refine the classification of SNPs at multicopy regions based on read-ratio deviations, when these are available in the input file. This increases the sensitivity to detect mid- to low-frequency SNPs at multicopy regions, which are often missed based on excess heterozygosity and classified as uncertain in the previous step. For this, we compute z-scores for read-ratio deviations at each SNP across individuals (Additional file 1: Supplementary Text). Different from previous approaches, instead of assuming a known null distribution for z-scores, we use the empirical distribution at single-copy SNPs classified in the previous step to construct two-sided 95% confidence intervals. This can account for overdispersion of empirical distributions compared to theoretical expectations. SNPs that were unclassified in the previous step, and with a z-score that lies outside the confidence interval are classified as SNPs at multicopy regions.

### Multicopy haplotypes are identified based on clustering of collapsed SNPs and excess sequencing depth

In the final step of the method, we combine the signatures of excess heterozygosity (modeled in the first step), read-ratio deviations (second step), excess depth, and proximity among collapsed SNPs, to identify multicopy haplotypes. The high-confidence multicopy SNPs identified in the first two steps tag multicopy haplotypes. Starting at these SNPs, and using them as seeds, we elongate multicopy haplotypes until we identify breakpoints with the adjacent single-copy regions. Initially, each collapsed SNP (seed) is assigned to a different multicopy region. If a neighboring SNP (focal SNP) down- or upstream of the seed is also a high-confidence multicopy SNP, the SNPs are concatenated into a merged multicopy region. If the neighboring SNP is uncertain or has strong support of belonging to a single-copy region, two scenarios are possible. First, between the seed and the focal SNP a breakpoint might exist between the multicopy haplotype and the adjacent single-copy region. In this case we expect no excess depth at the focal SNP across genotypes. Second, if the multicopy region is not fixed in the population, variation at the focal SNP might be located on a single-copy haplotype (e.g., a mutation on the left-most branch in Fig. 1a). In this case, the SNP would not be a seed, but would be present within the boundaries of the multicopy haplotype that segregates in other individuals. These individuals are expected to have excess depth. To distinguish between these cases, we analyze genotype-specific depth at the focal SNP and genome-wide. The individuals that are heterozygote at the nearest seed carry the multicopy haplotype. If these individuals have excess depth at the focal SNP (a minimum of 1.5 times the genomic average), we concatenate the focal SNP into a merged multicopy region. If the focal SNP does not have excess depth, and is classified as single-copy, we define a breakpoint between a single-copy and a multicopy region at the mid-distance between the previous SNP and the focal SNP. If the focal SNP does not have excess depth, and is classified as uncertain, we consider this case unclear and we assess the next neighboring SNP. Because in this case we are assessing SNPs at longer distances from the last seed, we additionally constrain the elongation of multicopy regions based on the distance among SNPs. Multicopy SNPs are clustered in haplotypes, therefore the distances among them are a mixture of distances within and between multicopy regions. We use an Expectation-Maximization approach (distinct from the first step of ParaMask) to estimate parameters of the two distributions and to define a threshold for distances within haplotypes (Additional file 1: Supplementary Text). If the average distance between the last seed, the focal SNP and the next neighboring SNP is shorter than the threshold, we assess this SNP in the same way described above. Otherwise, we define a breakpoint between a multicopy and a single-copy region at the mid-distance between the last seed and its neighboring SNP. When the mid-distance exceeds half of the distance threshold, we set the breakpoint to half the distance threshold from the last seed.

### Simulations

We simulated genomes with single-copy regions interspersed with duplications using the forward-in-time simulator of segmental duplications SeDuS [60]. We independently simulated single-copy and duplicated haplotypes, and then we collated them into a sequence of length approximately 1 Mbp. Details are described in Additional file 1: Supplementary Text. We simulated sequencing reads using ART Illumina *v*2.5.8 [61].

### Plant material, sequencing and variant calling

We collected cuttings and seeds from 85 individual plants in two populations of *Arabis alpina* from Spain, for a total of 36 individuals from population ES03 and 49 individuals from population ES04 (Additional file 3: Table S2). We extracted DNA and sequenced whole genomes with 150 bp paired end short reads nano ball sequencing. SNP calling was performed using the GATK pipeline *v*4.2.0 [62], and the reference genome *Arabis alpina v5.1* [63]. More details on DNA extraction, sequencing, and SNP calling can be found in the Additional file 1: Supplementary Text.

We sequenced one accession from each of the two populations, ES03-014 and ES04-014, with PacBio Hifi long-reads at the Max Planck Genome Centre Cologne. To identify multicopy regions in the long reads we took two approaches. First, we aligned the long reads to the reference genome, and we called structural variants with sniffles2 *v2.2* [64] and cuteSV *v2.0.2* [65]. We considered high-confidence multicopy regions the duplications that were identified by both methods, and high-confidence single-copy regions where neither cuteSV nor sniffles2 called any SV. Second, we assembled the long-reads genomes de novo with Hifiasm *v0.19* [66] and Flye *2.9* [67], and scaffolded them with RagTag *v2.1.0* [68]. The assemblies were then evaluated for quality and completeness, and screened for interspersed repeats and low complexity DNA. More details on long reads sequencing, SV calling, and genome assemblies are given in Additional file 1: Supplementary Text [69–79].

### Genomic summary statistics and demographic inference

We computed genomic summary statistics in simulated and empirical data using hierfstat [80], VCFtools [81], and custom scripts available at https://github.com/Fulgione-group/ParaMask_analysis (Additional file 1: Supplementary Text). To infer demographic histories, we used dadi [47], and the jAFS with and without the multicopy regions identified with ParaMask. The best supported model for each jAFS was chosen according to the Akaike Information Criterion. More detail can be found in Additional file 1: Supplementary Text.

### Experimental design and statistics

For the synthetic data set, we simulated genomic data for 100 diploid individuals in three independent replicates for every combination of parameters. To evaluate performance of different methods on simulated data, we reported the average recall (defined as $true\ positives/(true\ positives + false\ negatives)$) and range across replicates for all SNPs, and separately for single-copy and multicopy SNPs. We note that recall at single-copy SNPs is directly linked to specificity ($true\ positives/(true\ positives + false\ positives)$) of multicopy SNPs, and vice versa, since false negatives in one category correspond to false positives in the other. To quantify the effect of multicopy regions on genomic summary statistics in simulations, we reported average values, and the range across replicates. In particular, for comparisons of diversity estimates we reported the mean and range of the fold-change. For comparisons of Tajima’s D and $F_{IS}$ we reported absolute differences and differences normalized by the empirical standard deviation at single-copy regions. In comparisons of summary statistics with and without multicopy regions we reported fold-changes of diversity estimates, absolute differences in $F_{IS}$, and Tajima’s *D* for each population, and relative differences in $F_{ST}$ between populations. The overlap between regions classified with ParaMask, rCNV and ngsParalog, and structural variants called from long reads was reported as the median across regions of the proportion of SNPs of the correct category (single-copy or multicopy). In comparisons between ParaMask and rCNV or ngsParalog we reported the overlap in each category, corresponding to a two-by-two contingency table. The description of statistical modeling for the classification of SNPs at the core of the ParaMask method is described in the previous “Methods” section and in Additional file 1: Supplementary Text.

### Software tool requirements

The ParaMask software requires java (*v*1.8), R ($\ge v4.04$), and R packages VGAM (*v*1.1.1) [59], ggplot2 ($\ge v3.4.0$) [82], patchwork ($\ge v1.0.0$) [83] and data.table ($\ge v1.12.8$) [84].

## Supplementary Information

Additional file 1: Supplementary Text. This file includes supplementary results and methods.

Additional file 2: Supplementary Figures. This file includes Figures S1 to S14.

Additional file 3: Supplementary Tables. This file includes Tables S1 to S14.

## Data Availability

The ParaMask method is available in GitHub under a GPL-3.0 license at: https://github.com/Fulgione-group/ParaMask [85]. All code used in data analyses and visualization is available in GitHub under a GPL-3.0 license at: https://github.com/Fulgione-group/ParaMask_analysis [86]. The sequencing dataset supporting the conclusions of this article is available in the European Nucleotide Archive (ENA) under the accession number PRJEB73825 [87]. The unfiltered VCF file of *O. gorbuscha* is publicly available in Dryad at https://datadryad.org/dataset/doi:10.5061/dryad.0vt4b8h2w [33] and the reference genome assembly is available in the NCBI sequence repository under the BioProject accession PRJNA556728 [88]. The VCF files and linkage maps for *L. sinapis* are available in Dryad at 10.5061/dryad.qrfj6q5m7 [34]. The VCF for Chinook salmon RAD-seq data is available at https://doi.org/10.5061/dryad.cm08m[27, 43], and the annotation of copy number status is available in Table S1.1 from McKinney et al. [44]. All data supporting conclusions, that are not included in the additional files, as well as a copy of the ParaMask method and scripts for analyses are available under a GPL-3.0 license in Zenodo at: https://doi.org/10.5281/zenodo.10892193 [89].

## References

[1] Treangen TJ, Salzberg SL. Repetitive DNA and next-generation sequencing: computational challenges and solutions. Nat Rev Genet. 2011;13(1):36–46. 10.1038/nrg3117. 22124482 10.1038/nrg3117 PMC3324860

[2] Hartasánchez DA, Brasó-Vives M, Heredia-Genestar JM, Pybus M, Navarro A. Effect of collapsed duplications on diversity estimates: what to expect. Genome Biol Evol. 2018;10(11):2899–905. 10.1093/gbe/evy223. 30364947 10.1093/gbe/evy223 PMC6239678

[3] O’Leary SJ, Puritz JB, Willis SC, Hollenbeck CM, Portnoy DS. These aren’t the loci you’re looking for: Principles of effective SNP filtering for molecular ecologists. Mol Ecol. 2018. 10.1111/mec.14792. 10.1111/mec.14792 29987880

[4] Vollger MR, Guitart X, Dishuck PC, Mercuri L, Harvey WT, Gershman A, et al. Segmental duplications and their variation in a complete human genome. Science. 2022;376(6588):eabj6965. 10.1126/science.abj6965. 35357917 10.1126/science.abj6965 PMC8979283

[5] Jaegle B, Pisupati R, Soto-Jiménez LM, Burns R, Rabanal FA, Nordborg M. Extensive sequence duplication in Arabidopsis revealed by pseudo-heterozygosity. Genome Biol. 2023;24(1):44. 10.1186/s13059-023-02875-3. 36895055 10.1186/s13059-023-02875-3 PMC9999624

[6] Chakraborty M, Chang CH, Khost DE, Vedanayagam J, Adrion JR, Liao Y, et al. Evolution of genome structure in the *Drosophila simulans* species complex. Genome Res. 2021;31(3):380–96. 10.1101/gr.263442.120. 33563718 10.1101/gr.263442.120 PMC7919458

[7] Jiao Y, Wickett NJ, Ayyampalayam S, Chanderbali AS, Landherr L, Ralph PE, et al. Ancestral polyploidy in seed plants and angiosperms. Nature. 2011;473(7345):97–100. 10.1038/nature09916. 21478875 10.1038/nature09916

[8] One Thousand Plant Transcriptomes Initiative. One thousand plant transcriptomes and the phylogenomics of green plants. Nature. 2019;574(7780):679–85. 10.1038/s41586-019-1693-2. 31645766 10.1038/s41586-019-1693-2 PMC6872490

[9] McLysaght A, Hokamp K, Wolfe KH. Extensive genomic duplication during early chordate evolution. Nat Genet. 2002;31(2):200–4. 10.1038/ng884. 12032567 10.1038/ng884

[10] Dehal P, Boore JL. Two rounds of whole genome duplication in the ancestral vertebrate. PLoS Biol. 2005;3(10):e314. 10.1371/journal.pbio.0030314. 16128622 10.1371/journal.pbio.0030314 PMC1197285

[11] Berthelot C, Brunet F, Chalopin D, Juanchich A, Bernard M, Noël B, et al. The rainbow trout genome provides novel insights into evolution after whole-genome duplication in vertebrates. Nat Commun. 2014;5:3657. 10.1038/ncomms4657. 24755649 10.1038/ncomms4657 PMC4071752

[12] Li Z, Tiley GP, Galuska SR, Reardon CR, Kidder TI, Rundell RJ, et al. Multiple large-scale gene and genome duplications during the evolution of hexapods. Proc Natl Acad Sci U S A. 2018;115(18):4713–8. 10.1073/pnas.1710791115. 29674453 10.1073/pnas.1710791115 PMC5939055

[13] Wolfe KH, Shields DC. Molecular evidence for an ancient duplication of the entire yeast genome. Nature. 1997;387(6634):708–13. 10.1038/42711. 9192896 10.1038/42711

[14] Tenaillon MI, Hollister JD, Gaut BS. A triptych of the evolution of plant transposable elements. Trends Plant Sci. 2010;15(8):471–8. 10.1016/j.tplants.2010.05.003. 20541961 10.1016/j.tplants.2010.05.003

[15] Sproul JS, Hotaling S, Heckenhauer J, Powell A, Marshall D, Larracuente AM, et al. Analyses of 600+ insect genomes reveal repetitive element dynamics and highlight biodiversity-scale repeat annotation challenges. Genome Res. 2023;33(10):1708–17. 10.1101/gr.277387.122. 37739812 10.1101/gr.277387.122 PMC10691545

[16] Sun C, Shepard DB, Chong RA, López Arriaza J, Hall K, Castoe TA, et al. Ltr retrotransposons contribute to genomic gigantism in plethodontid salamanders. Genome Biol Evol. 2012;4(2):168–83. 10.1093/gbe/evr139. 22200636 10.1093/gbe/evr139 PMC3318908

[17] Wang K, Wang J, Zhu C, Yang L, Ren Y, Ruan J, et al. African lungfish genome sheds light on the vertebrate water-to-land transition. Cell. 2021;184(5):1362-1376.e18. 10.1016/j.cell.2021.01.047. 33545087 10.1016/j.cell.2021.01.047

[18] Brasó-Vives M, Marlétaz F, Echchiki A, Mantica F, Acemel RD, Gómez-Skarmeta JL, et al. Parallel evolution of amphioxus and vertebrate small-scale gene duplications. Genome Biol. 2022;23(1):243. 10.1186/s13059-022-02808-6. 36401278 10.1186/s13059-022-02808-6 PMC9673378

[19] Ohno S. Evolution by gene duplication. London, New York: Allen & Unwin; Springer-Verlag; 1970.

[20] Zhang J. Evolution by gene duplication: an update. Trends Ecol Evol. 2003;18(6):292–298. Publisher: Elsevier.

[21] Conant GC, Wolfe KH. Turning a hobby into a job: how duplicated genes find new functions. Nat Rev Genet. 2008;9(12):938–50. 10.1038/nrg2482. 19015656 10.1038/nrg2482

[22] Hanikenne M, Talke IN, Haydon MJ, Lanz C, Nolte A, Motte P, et al. Evolution of metal hyperaccumulation required *cis*-regulatory changes and triplication of *HMA4*. Nature. 2008;453(7193):391–5. 10.1038/nature06877. 18425111 10.1038/nature06877

[23] Tergemina E, Elfarargi AF, Flis P, Fulgione A, Göktay M, Neto C, et al. A two-step adaptive walk rewires nutrient transport in a challenging edaphic environment. Sci Adv. 2022;8(20):eabm9385. 10.1126/sciadv.abm9385. 35584228 10.1126/sciadv.abm9385 PMC9116884

[24] Willis SC, Hollenbeck CM, Puritz JB, Gold JR, Portnoy DS. Haplotyping RAD loci: an efficient method to filter paralogs and account for physical linkage. Mol Ecol Resour. 2017;17(5):955–65. 10.1111/1755-0998.12647. 28042915 10.1111/1755-0998.12647

[25] Gutiérrez-Valencia J, Fracassetti M, Horvath R, Laenen B, Désamore A, Drouzas AD, et al. Genomic signatures of sexual selection on pollen-expressed genes in *Arabis alpina*. Mol Biol Evol. 2022;39(1):msab349. 10.1093/molbev/msab349. 34878144 10.1093/molbev/msab349 PMC8788238

[26] Fulgione A, Neto C, Elfarargi AF, Tergemina E, Ansari S, Göktay M, et al. Parallel reduction in flowering time from de novo mutations enable evolutionary rescue in colonizing lineages. Nat Commun. 2022;13(1):1461. 10.1038/s41467-022-28800-z. 35304466 10.1038/s41467-022-28800-z PMC8933414

[27] McKinney GJ, Waples RK, Seeb LW, Seeb JE. Paralogs are revealed by proportion of heterozygotes and deviations in read ratios in genotyping-by-sequencing data from natural populations. Mol Ecol Resour. 2017;17(4):656–69. 10.1111/1755-0998.12613. 27762098 10.1111/1755-0998.12613

[28] Catchen J, Hohenlohe PA, Bassham S, Amores A, Cresko WA. Stacks: an analysis tool set for population genomics. Mol Ecol. 2013;22(11):3124–40. 10.1111/mec.12354. 23701397 10.1111/mec.12354 PMC3936987

[29] Dorant Y, Cayuela H, Wellband K, Laporte M, Rougemont Q, Mérot C, et al. Copy number variants outperform SNPs to reveal genotype-temperature association in a marine species. Mol Ecol. 2020;29(24):4765–82. 10.1111/mec.15565. 32803780 10.1111/mec.15565

[30] Karunarathne P, Zhou Q, Schliep K, Milesi P. A comprehensive framework for detecting copy number variants from single nucleotide polymorphism data: ‘rCNV’, a versatile r package for paralogue and CNV detection. Mol Ecol Resour. 2023;23(8):1772–89. 10.1111/1755-0998.13843. 37515483 10.1111/1755-0998.13843

[31] Linderoth T. In: Identifying population histories, adaptive genes, and genetic duplication from population-scale next generation sequencing. Doctoral dissertation, Berkeley: University of California; 2018. pp. 5–39. https://escholarship.org/uc/item/5kp4q40k. Accessed 07 Aug 2025.

[32] Waples RS. Testing for Hardy-Weinberg proportions: have we lost the plot? J Hered. 2015;106(1):1–19. 10.1093/jhered/esu062. 25425676 10.1093/jhered/esu062

[33] Sparks MM, Schraidt CE, Yin X, Seeb LW, Christie MR. Rapid genetic adaptation to a novel ecosystem despite a large founder event. Mol Ecol. 2023;1–16. 10.1111/mec.17121. 10.1111/mec.17121 PMC13084982 37668092

[34] Torres AP, Höök L, Näsvall K, Shipilina D, Wiklund C, Vila R, et al. The fine-scale recombination rate variation and associations with genomic features in a butterfly. Genome Res. 2023;33(5):810–23. 10.1101/gr.277414.122. 37308293 10.1101/gr.277414.122 PMC10317125

[35] Macqueen DJ, Johnston IA. A well-constrained estimate for the timing of the salmonid whole genome duplication reveals major decoupling from species diversification. Proc R Soc Lond B Biol Sci. 2014. 10.1098/rspb.2013.2881. 10.1098/rspb.2013.2881 PMC3906940 24452024

[36] Ohno S, Wolf U, Atkin NB. Evolution from fish to mammals. Hereditas. 1968;59(1):169–87. 5662632 10.1111/j.1601-5223.1968.tb02169.x

[37] Allendorf FW, Thorgaard GH. In: Turner BJ, editor. Tetraploidy and the evolution of salmonid fishes. Boston: Springer US; 1984. pp. 1–53. 10.1007/978-1-4684-4652-4_1.

[38] Brieuc MSO, Waters CD, Seeb JE, Naish KA. A dense linkage map for chinook salmon (*Oncorhynchus tshawytscha*) reveals variable chromosomal divergence after an ancestral whole genome duplication event. G3 Genes|Genomes|Genetics. 2014;4(3):447–60. 10.1534/g3.113.009316. 24381192 10.1534/g3.113.009316 PMC3962484

[39] Kodama M, Brieuc MSO, Devlin RH, Hard JJ, Naish KA. Comparative mapping between coho salmon (*Oncorhynchus kisutch*) and three other salmonids suggests a role for chromosomal rearrangements in the retention of duplicated regions following a whole genome duplication event. G3 Genes Genomes Genet. 2014;9:1717–30. 10.1534/g3.114.012294. 10.1534/g3.114.012294 PMC4169165 25053705

[40] Laenen B, Tedder A, Nowak MD, Toräng P, Wunder J, Wötzel S, et al. Demography and mating system shape the genome-wide impact of purifying selection in *Arabis alpina*. Proc Natl Acad Sci U S A. 2018;115(4):816–21. 29301967 10.1073/pnas.1707492115 PMC5789905

[41] Alexa A, Rahnenfuehrer J, Lengauer T. Improved scoring of functional groups from gene expression data by decorrelating GO graph structure. Bioinformatics. 2006;22(13):1600–7. 10.1093/bioinformatics/btl140 16606683

[42] Dallaire X, Bouchard R, Hénault P, Ulmo-Diaz G, Normandeau E, Mérot C, et al. Widespread deviant patterns of heterozygosity in whole-genome sequencing due to autopolyploidy, repeated elements, and duplication. Genome Biol Evol. 2023;15(12):evad229. 10.1093/gbe/evad229. 38085037 10.1093/gbe/evad229 PMC10752349

[43] Larson WA, Seeb LW, Everett MV, Waples RK, Templin WD, Seeb JE. Genotyping by sequencing resolves shallow population structure to inform conservation of Chinook salmon (*Oncorhynchus tshawytscha*). Evol Appl. 2014;7(3):355–69. 24665338 10.1111/eva.12128 PMC3962296

[44] McKinney G, Seeb L, Larson W, Gomez-Uchida D, Limborg MT, Brieuc M, et al. An integrated linkage map reveals candidate genes underlying adaptive variation in Chinook salmon (*Oncorhynchus tshawytscha*). Mol Ecol Resour. 2016;16(3):769–83. 26490135 10.1111/1755-0998.12479

[45] Watterson GA. On the number of segregating sites in genetical models without recombination. Theor Popul Biol. 1975;7(2):256–76. 10.1016/0040-5809(75)90020-9. 1145509 10.1016/0040-5809(75)90020-9

[46] Tajima F. Statistical method for testing the neutral mutation hypothesis by DNA polymorphism. Genetics. 1989;123(3):585–95. 10.1093/genetics/123.3.585. 2513255 10.1093/genetics/123.3.585 PMC1203831

[47] Gutenkunst RN, Hernandez RD, Williamson SH, Bustamante CD. Inferring the joint demographic history of multiple populations from multidimensional SNP frequency data. PLoS Genet. 2009;5(10):e1000695. 10.1371/journal.pgen.1000695. 19851460 10.1371/journal.pgen.1000695 PMC2760211

[48] Thornton KR. The neutral coalescent process for recent gene duplications and copy-number variants. Genetics. 2007;177(2):987–1000. 10.1534/genetics.107.074948. 17720930 10.1534/genetics.107.074948 PMC2034660

[49] Willing EM, Rawat V, Mandáková T, Maumus F, James GV, Nordström KJV, et al. Genome expansion of *Arabis alpina* linked with retrotransposition and reduced symmetric DNA methylation. Nat Plants. 2015;1(2):14023. 10.1038/nplants.2014.23. 27246759 10.1038/nplants.2014.23

[50] Teshima KM, Innan H. The effect of gene conversion on the divergence between duplicated genes. Genetics. 2004;166(3):1553–60. 10.1534/genetics.166.3.1553. 15082568 10.1534/genetics.166.3.1553 PMC1470786

[51] Wolf JBW, Ellegren H. Making sense of genomic islands of differentiation in light of speciation. Nat Rev Genet. 2017;18(2):87–100. 10.1038/nrg.2016.133. 27840429 10.1038/nrg.2016.133

[52] Charlesworth D. Balancing selection and its effects on sequences in nearby genome regions. PLoS Genet. 2006;2(4):e64. 10.1371/journal.pgen.0020064. 16683038 10.1371/journal.pgen.0020064 PMC1449905

[53] Bitarello BD, Brandt DYC, Meyer D, Andrés AM. Inferring balancing selection from genome-scale data. Genome Biol Evol. 2023;15(3):evad032. 10.1093/gbe/evad032. 36821771 10.1093/gbe/evad032 PMC10063222

[54] Hughes AL, Yeager M. Natural selection at major histocompatibility complex loci of vertebrates. Annu Rev Genet. 1998;32(1):415–35. 10.1146/annurev.genet.32.1.415. 9928486 10.1146/annurev.genet.32.1.415

[55] Radwan J, Babik W, Kaufman J, Lenz TL, Winternitz J. Advances in the evolutionary understanding of MHC polymorphism. Trends Genet. 2020;36(4):298–311. 10.1016/j.tig.2020.01.008. 32044115 10.1016/j.tig.2020.01.008

[56] Bergelson J, Kreitman M, Stahl EA, Tian D. Evolutionary dynamics of plant R-genes. Science. 2001;292(5525):2281–5. 10.1126/science.1061337. 11423651 10.1126/science.1061337

[57] Wu J, Saupe SJ, Glass NL. Evidence for balancing selection operating at the het-c heterokaryon incompatibility locus in a group of filamentous fungi. Proc Natl Acad Sci USA. 1998;95(21):12398–403. 10.1073/pnas.95.21.12398. 9770498 10.1073/pnas.95.21.12398 PMC22843

[58] Madrid E, Severing E, de Ansorena E, Kiefer C, Brand L, Martinez-Gallegos R, et al. Transposition and duplication of MADS-domain transcription factor genes in annual and perennial *Arabis* species modulates flowering. Proc Natl Acad Sci U S A. 2021;118(39):e2109204118. 10.1073/pnas.2109204118. 34548402 10.1073/pnas.2109204118 PMC8488671

[59] Yee TW, Wild CJ. Vector generalized additive models. J Roy Stat Soc: Ser B (Methodol). 1996;58(3):481–93. 10.1111/j.2517-6161.1996.tb02095.x.

[60] Hartasánchez DA, Brasó-Vives M, Fuentes-Díaz J, Vallès-Codina O, Navarro A. SeDuS: segmental duplication simulator. Bioinformatics (Oxford England). 2016;32(1):148–50. 10.1093/bioinformatics/btv481. 26358728 10.1093/bioinformatics/btv481

[61] Huang W, Li L, Myers JR, Marth GT. ART: a next-generation sequencing read simulator. Bioinformatics. 2012;28(4):593–4. 22199392 10.1093/bioinformatics/btr708 PMC3278762

[62] DePristo MA, Banks E, Poplin R, Garimella KV, Maguire JR, Hartl C, et al. A framework for variation discovery and genotyping using next-generation DNA sequencing data. Nat Genet. 2011;43(5):491–8. 10.1038/ng.806. 21478889 10.1038/ng.806 PMC3083463

[63] Jiao WB, Accinelli GG, Hartwig B, Kiefer C, Baker D, Severing E, et al. Improving and correcting the contiguity of long-read genome assemblies of three plant species using optical mapping and chromosome conformation capture data. Genome Res. 2017;27(5):778–86. 10.1101/gr.213652.116. 28159771 10.1101/gr.213652.116 PMC5411772

[64] Smolka M, Paulin LF, Grochowski CM, Horner DW, Mahmoud M, Behera S, et al. Detection of mosaic and population-level structural variants with Sniffles2. Nat Biotechnol. 2024;42:1571–80. 10.1038/s41587-023-02024-y PMC11217151 38168980

[65] Jiang T, Liu Y, Jiang Y, Li J, Gao Y, Cui Z, et al. Long-read-based human genomic structural variation detection with cuteSV. Genome Biol. 2020;21(1):1–24. 10.1186/s13059-020-02107-y PMC7477834 32746918

[66] Cheng H, Concepcion GT, Feng X, Zhang H, Li H. Haplotype-resolved de novo assembly using phased assembly graphs with hifiasm. Nat Methods. 2021;18(2):170–5. 33526886 10.1038/s41592-020-01056-5 PMC7961889

[67] Kolmogorov M, Yuan J, Lin Y, Pevzner PA. Assembly of long, error-prone reads using repeat graphs. Nat Biotechnol. 2019;37(5):540–6. 30936562 10.1038/s41587-019-0072-8

[68] Alonge M, Lebeigle L, Kirsche M, Jenike K, Ou S, Aganezov S, et al. Automated assembly scaffolding using RagTag elevates a new tomato system for high-throughput genome editing. Genome Biol. 2022;23(1):1–19. 36522651 10.1186/s13059-022-02823-7 PMC9753292

[69] Li H. Minimap2: pairwise alignment for nucleotide sequences. Bioinformatics. 2018;34(18):3094–100. 29750242 10.1093/bioinformatics/bty191 PMC6137996

[70] Ranallo-Benavidez TR, Jaron KS, Schatz MC. GenomeScope 2.0 and Smudgeplot for reference-free profiling of polyploid genomes. Nat Commun. 2020;11(1):1432. 32188846 10.1038/s41467-020-14998-3 PMC7080791

[71] Marla SS, Mishra P, Maurya R, Singh M, Wankhede DP, Kumar A, et al. Refinement of draft genome assemblies of pigeonpea (*Cajanus cajan*). Front Genet. 2020;11:607432. 33384719 10.3389/fgene.2020.607432 PMC7770131

[72] Shumate A, Salzberg SL. Liftoff: accurate mapping of gene annotations. Bioinformatics. 2021;37(12):1639–43. 33320174 10.1093/bioinformatics/btaa1016 PMC8289374

[73] Mikheenko A, Prjibelski A, Saveliev V, Antipov D, Gurevich A. Versatile genome assembly evaluation with QUAST-LG. Bioinformatics. 2018;34(13):i142-50. 29949969 10.1093/bioinformatics/bty266 PMC6022658

[74] Simão FA, Waterhouse RM, Ioannidis P, Kriventseva EV, Zdobnov EM. BUSCO: assessing genome assembly and annotation completeness with single-copy orthologs. Bioinformatics. 2015;31(19):3210–2. 26059717 10.1093/bioinformatics/btv351

[75] Smit A, Hubley R, Green P. RepeatMasker Open-4.0. RMDownload html. 2013. https://www.repeatmasker.org. Accessed 26 Jan 2024.

[76] Guan D, McCarthy SA, Wood J, Howe K, Wang Y, Durbin R. Identifying and removing haplotypic duplication in primary genome assemblies. Bioinformatics. 2020;36(9):2896–8. 31971576 10.1093/bioinformatics/btaa025 PMC7203741

[77] Jeffares DC, Jolly C, Hoti M, Speed D, Shaw L, Rallis C, et al. Transient structural variations have strong effects on quantitative traits and reproductive isolation in fission yeast. Nat Commun. 2017;8(1):14061. 28117401 10.1038/ncomms14061 PMC5286201

[78] Marçais G, Kingsford C. A fast, lock-free approach for efficient parallel counting of occurrences of k-mers. Bioinformatics. 2011;27(6):764–70. 21217122 10.1093/bioinformatics/btr011 PMC3051319

[79] Chakraborty M, Baldwin-Brown JG, Long AD, Emerson J. Contiguous and accurate de novo assembly of metazoan genomes with modest long read coverage. Nucleic Acids Res. 2016;44(19):e147. 27458204 10.1093/nar/gkw654 PMC5100563

[80] Goudet J. Hierfstat, a package for r to compute and test hierarchical F-statistics. Mol Ecol Notes. 2005;5(1):184–6. 10.1111/j.1471-8286.2004.00828.x.

[81] Danecek P, Auton A, Abecasis G, Albers CA, Banks E, DePristo MA, et al. The variant call format and VCFtools. Bioinformatics. 2011;27(15):2156–8. 10.1093/bioinformatics/btr330. 21653522 10.1093/bioinformatics/btr330 PMC3137218

[82] Wickham H. ggplot2: elegant graphics for data analysis. New York: Springer-Verlag; 2016. ISBN: 978-3-319-24277-4.

[83] Pedersen TL. patchwork: The Composer of Plots. 2024. R package version 1.2.0.9000. https://github.com/thomasp85/patchwork. Accessed 10 June 2024.

[84] Barrett T, Dowle M, Srinivasan A, Gorecki J, Chirico M, Hocking T. data.table: Extension of ‘data.frame’. 2024. R package version 1.15.99. https://github.com/Rdatatable/data.table. Accessed 10 June 2024.

[85] Tjeng B, Arimond M, Dalla Libera A, Grindeland HB, Fulgione A. ParaMask: a new method to identify multicopy genomic regions, corrects major biases in whole-genome sequencing data. ParaMask Method. Cologne: GitHub; 2024. https://github.com/Fulgione-group/ParaMask. Accessed 14 Sept 2025. 10.1186/s13059-025-03836-8 PMC12551310 41137035

[86] Tjeng B, Arimond M, Dalla Libera A, Grindeland HB, Fulgione A. ParaMask: a new method to identify multicopy genomic regions, corrects major biases in whole-genome sequencing data. ParaMask Analysis. Cologne: GitHub; 2024. https://github.com/Fulgione-group/ParaMask_Analysis. Accessed 21 Aug 2025. 10.1186/s13059-025-03836-8 PMC12551310 41137035

[87] Tjeng B, Arimond M, Dalla Libera A, Grindeland HB, Fulgione A. ParaMask: a new method to identify multicopy genomic regions, corrects major biases in whole-genome sequencing data. Sequencing Datasets. Cologne: European Nucleotide Archive; 2024. https://www.ebi.ac.uk/ena/browser/view/PRJEB73825. Accessed 02 Oct 2025. 10.1186/s13059-025-03836-8 PMC12551310 41137035

[88] Christensen KA, Rondeau EB, Sakhrani D, Biagi CA, Johnson H, Joshi J, et al. The pink salmon genome: uncovering the genomic consequences of a two-year life cycle. PLoS ONE. 2021;16(12 December):1–33. 10.1371/journal.pone.0255752. 10.1371/journal.pone.0255752 PMC8682878 34919547

[89] Tjeng B, Arimond M, Dalla Libera A, Grindeland HB, Fulgione A. ParaMask: a new method to identify multicopy genomic regions, corrects major biases in whole-genome sequencing data. Additional Datasets. Cologne: Zenodo; 2024. 10.5281/zenodo.10892193. Accessed 21 Aug 2025. 10.1186/s13059-025-03836-8 PMC12551310 41137035

