# Supplementary material for: ParaMask: a new method to identify multicopy genomic regions, corrects major biases in whole-genome sequencing data
Source: Genome Biol. 2025 Oct 24;26:368. doi: 10.1186/s13059-025-03836-8 (PMC12551310; doi:10.1186/s13059-025-03836-8)
Supplement: Supplementary file 1 — Additional file 1: Supplementary Text. This file includes supplementary results and methods. [file 13059_2025_3836_MOESM1_ESM.pdf]

ParaMask: a new method to identify multicopy genomic regions, corrects major biases in whole-genome sequencing data

Additional file 1: Supplementary Text

Bastiaan Tjeng[1], Male Arimond[1], Helene Bråten Grindeland[1],

Andrea Dalla Libera[1], Andrea Fulgione[1,*]

October 6, 2025

[1]: Max Planck Institute for Plant Breeding Research, Carl-von-Linne-Weg 10, 50829, Cologne

***Corresponding author:** Andrea Fulgione, e-mail: fulgione@mpipz.mpg.de

Contacts:

Bastiaan Tjeng: btjeng@mpipz.mpg.de

Male Arimond: marimond@mpipz.mpg.de

Helene Bråten Grindeland: hgrindeland@mpipz.mpg.de

Andrea Dalla Libera: adallalibera@mpipz.mpg.de

Andrea Fulgione: fulgione@mpipz.mpg.de

An Expectation-Maximization approach to identify single-copy and multicopy regions from heterozygosity levels

The joint regression of heterozygote frequencies consists on first duplicating the data set, and assigning one replicate to fit SNPs at multicopy regions (level D of factor Z), and the other to fit SNPs at single-copy regions (level K of factor Z). Across the two replicates, every SNP (x_i) is associated with a weight (W_{x_i}) between [0; 1]. Weights are used as soft assignments, and are equivalent to a quantitative measure of the posterior likelihood for a SNP to belong to single-copy, or to multicopy regions. The weights in the two replicate data sets are linked, so that: $W_{x_i|Z==D} = 1 - W_{x_i|Z==K}$. At the initialization of the model, we assign a weight of 0.01 to SNPs with heterozygote frequency $f(Aa) = 2 * maf$ and to all SNPs with transformed heterozygote frequencies that fall into the 0.1% upper tail of the binomial distribution when assuming Hardy-Weinberg proportions ($E[f(Aa)/2maf] = 1 - maf$). We assign a weight of 0.99 to the SNPs that fall in the 0.1% lower tail of that distribution. To all other SNPs we assign weights randomly from a uniform distribution between 0.01 and 0.99. This moderately informed initialization strategy accelerates convergence and prevents suboptimal results. At every iteration step, we use data and weights to fit the mean via the Complementary Log-Log link and overdispersion via the logit link. For the mean we use the following model:

$$f(Aa) \sim Z + I((1 - maf) * (Z == K))$$

The model fits an intercept for each level of factor Z and an interaction term I of level K (corresponding to single-copy SNPs) with $(1 - maf)$. The intercept can converge towards $(1 - F_{IS})$ fitting the inbreeding coefficient to the data ($F_{IS} = 0$ with random mating). Because $2 * (1 - F_{IS})$ is independent of the maf, it is absorbed in the regression parameters for SNPs at single-copy regions. This allows the model to fit the inbreeding coefficient to the data, and to avoid assumptions on random mating and Hardy-Weinberg proportions of genotype frequencies. For multicopy regions, the model fits a constant hazard ratio for $f(Aa)/2maf$, with no interaction term, corresponding to a straight line. For the overdispersion we fit a single model with only one intercept for both levels, which results in equal overdispersion assigned to SNPs at single-copy and multicopy regions. This constrains the model, while avoiding assumptions about the relative effect of the various sources of overdispersion.

After fitting we calculate probability densities from predicted means for SNPs at single-copy and at multicopy regions with the dbetabinom() function. Then we recalibrate weights for each replicate data set as:

$$W_{x_i|Z=K} = \frac{P(Z=K) \cdot P(X=x_i \mid Z=K)}{P(Z=K) \cdot P(X=x_i \mid Z=K) + P(Z=D) \cdot P(X=x_i \mid Z=D)}$$

Where $P(Z = K)$ and $P(Z = D)$ are given by the weigths of the previous iteration. We iterate these steps until likelihood convergence is reached, or for a user-specified number of iterations.

The probability densities from the last iteration of the EM algorithm are a measure of the likelihood (L) of the SNP belonging to the factor Z (whether a SNP belongs to a single-copy or to a multicopy region) given the observed heterozygous frequencies at the focal SNP and genomewide. We use these probabilities to obtain the log-likelihood ratio (λ) as a measure of the relative support for a model where the SNP originated at a single-copy or at a multicopy region. We classify SNPs as multicopy SNPs when $\lambda \leq log(L_{Z==K} = 0.01/L_{Z==D} = 0.99) \leq -4.59512$, and as single-copy SNPs when $\lambda \geq log(L_{Z==K} = 0.99/L_{Z==D} = 0.01) \geq 4.59512$). The remaining SNPs are considered of uncertain origin (when $-4.59512 \leq \lambda \leq 4.59512$), and they include many low-frequency SNPs where expectations for single-copy and multicopy regions converge (supplementary fig. S1b).

Read-ratio deviations

We calculated read-ratio deviations based on a z-score at each SNP across individuals according to [1], so that:

$$z_i = \frac{p * N_i - Na_i}{\sqrt{N_i * p * (1-p)}}$$

Where N_i is the total read depth at a SNP i, Na_i is the read depth of the alternative allele and p is the expected read ratio of 0.5. Different from previous approaches, we construct a two-sided 95% normal confidence interval based on the mean and variance of the empirical z-score distribution at single-copy SNPs classified in the first EM-based step. This can account for unknown sources of variance that may increase overdispersion at empirical distributions compared to theoretical expectations. SNPs that were unclassified in the previous step, and with a z-score that lies outside the confidence interval are

classified as SNPs in multicopy regions in this step. Expected read ratios for heterozygote genotypes at single-copy regions is 0.5. However, sequencing technologies can have a reference bias, mainly caused by the difference in mapping accuracy of reads carrying additional mismatches. This happens especially in SNP calling pipelines where SNPs are not called directly from the alignment files, as for instance in GATK. For multicopy regions, expected read ratios depend on the number of copies and on the frequency of heterozygotes on each copy. For empirical data where we called SNPs with GATK, we note that this pipeline only counts 'informative reads' in the reported allele depth, while also uninformative reads might be involved in the genotype call. Informative reads are reads with a marginal likelihood difference to the second most likely allele of 0.2 on a Phred scale.

In the empirical data of *A. alpina*, SNPs classified as single-copy have a median read ratio of 0.5035 and a single mode at 0.5079 (supplementary fig. S4). These read ratios are very close to the expected 0.5, with a slight deviation that is likely due to reference bias. SNPs flagged as seeds in the EM step of ParaMask had a median read ratio of 0.6934 and a bimodal distribution with maxima at 0.5606 and 0.8129. The bimodal density of seeds may stem from the expected read ratio of 0.5 for homozygote differences between copies, and of 0.25 or 0.75 for sites that are heterozygote on one copy and homozygote on the other. Also, the presence of regions with more than two copies would result in different expected read ratios. The shifts of the modes might result from increased reference bias for multicopy regions due to decreased mapping accuracy of reads at the flanks of the multicopy region. SNPs that were of uncertain origin based on the EM, and that are mainly at low frequency, have median read ratio of 0.763158 and a bimodal density with maxima at 0.5017 and 0.8836. The two modes of the uncertain SNPs suggest that they are likely a mixture of SNPs at single-copy and at multicopy regions. The relationship between heterozygote frequency and read-ratio deviations is weaker in our data (supplementary fig. S4) compared to other studies on outcrossing species [1]. This is likely due to the low prevalence of heterozygotes due to selfing in *A. alpina*. As a consequence of this, SNPs in multicopy regions are not too well characterised by read-ratio deviations in inbred populations, consistent with our results on simulations and on empirical data.

Clustering of multicopy haplotypes

To compute the distance threshold for haplotype clustering, we assume that the distances between seed SNPs are randomly distributed within haplotypes and haplotypes are randomly distributed across the genome. Under these assumptions, the distances between seed SNPs are a mixture of a geometric distribution of distances within haplotypes, and one between haplotypes. Within ParaMask, we use an Expectation-Maximization approach to estimate parameters of the two distributions (this differs from the EM in the first step of ParaMask). We output as a threshold the distance at which the density of distances within haplotypes equals the density of distances among haplotypes. Because distances within haplotypes tend to be shorter than among haplotypes, distances that are larger than this threshold are more likely to reflect distances among than within haplotypes. To obtain a stable distance threshold that is less affected by randomness in the EM algorithm, we repeat this estimation procedure 1000 times by default and report the median across replicates. At the end of the clustering procedure, if a multicopy SNP is isolated in between single-copy SNPs, we consider it suspicious and we omit it from the final classification in multicopy haplotypes.

Simulations

We simulated genomes with single-copy regions interspersed with duplications using the forward-in-time simulator of segmental duplications SeDuS [2]. Every simulation outputs three blocks, corresponding to one single-copy haplotype, and two copies for a different duplicated haplotype. To approximate a genome with single-copy regions interspersed with duplications, we ran the simulations multiple times, then collated single-copy to duplicated haplotypes to reach a total unique sequence of approximate length 1 Mbp. For the duplications, we sampled haplotype lengths from an exponential distribution with average 1 kb, for an approximate total length of $0.1*1$ Mbp for simulations with 10% duplications, and $0.5 * 1$ Mbp for simulations with 50% duplications. Note that these are lengths after collapsing, therefore we effectively simulate twice these sequence lengths. For the simulations, we set population size at 10000 individuals, sample size at 100 individuals, and recombination rate at $5 * 10^{-7}$ per base pair per generation (adjusting the effective allelic recombination rate per chromosome). All other parameters were set at default values, including a mutation rate of $2.5*10^{-8}$ mutations per generation and

population-scale gene conversion rate of 0.05 per generation. To simulate single-copy and duplicated haplotypes in an inbred population we rescaled parameters in SeDus by dividing effective population size and mutation rate by $(1 + F_{IS})$ and by multiplying recombination rate by $(1 - F_{IS})$. An inbreeding coefficient of $F_{IS} = 0.9$ corresponds to a selfing rate of approximately $s = 0.95$. To approximate the expected genotype frequencies with inbreeding, we implemented an additional step, and converted original heterozygote genotypes to homozygous genotypes with probability equal to $F_{IS} = 0.9$. We note, that with this approximation we effectively reduce diversity further by approximately 9%. In order to recover the pattern expected when multicopy regions are collapsed, we kept track of each genotype (adjusted for heterozygosity in the case of inbreeding) at single-copy regions and at each copy of duplicated regions. For each SNP, we assigned sequencing depth by randomly sampling from a negative binomial distribution with overdispersion parameter $\theta_{NB} = 8$ and mean 10 for SNPs at single-copy regions, and mean 20 for SNPs at duplicated regions. In a second step, we assign read ratios based on the read depth assigned to each SNP. For homozygous genotypes, all reads are assigned to the only allele present. For heterozygous genotypes at single-copy regions and for collapsed duplications that are homozygote for different alleles, read ratios are sampled from a binomial distribution with mean 0.5, and given read depth. For genotypes that are heterozygous on one duplicated copy and homozygote on the other, read ratios are sampled from a binomial distribution with mean 0.25 for duplications with one derived allele, and with mean 0.75, for duplications with three derived alleles. We note that this latter case happens in cases of interlocus gene conversion. SNPs with a read ratio lower than 0.2 or higher than 0.8 are set as homozygotes. Genotypes with read depth less than three are set to missing values.

To evaluate the performance of ParaMask with different sequencing depths, we used simulated sequences from SeDuS with 10% duplications and we modeled a read depth of 3x, 5x, 8x, 12x, and 20x with a negative binomial model, with random mating ($F_{IS} = 0$) and inbreeding ($F_{IS} = 0.9$). In this case, genotypes were only set to missing values when depth was less than two. To assess the performance of ParaMask with different sample sizes we sub-sampled 10, 15, 20, and 50 genotypes from all simulations with different read depths. For analyses on all combinations of sample size and read depth, we excluded sites with more than 30% missing genotypes using the ParaMask parameter

$-m$. All other ParaMask parameters were kept at default values.

Identification of multicopy regions in simulations with low sample size and low sequencing depth

In ParaMask, the EM algorithm failed to converge for a sample size of 10 and random mating, and it did converge with inbreeding. We therefore focused on simulations with sample sizes of 15 and above. Across all combinations of sample size and read depth explored, recall for single-copy SNPs remained high with random mating and with inbreeding (above 99.0% on average; supplementary fig. S3). At multicopy regions, recall declined with smaller sample sizes and lower read depth but remained high with inbreeding, even at a minimal read depth of 3× and sample size of 15 (with a recall of 86.6% on average across replicates; supplementary fig. S3). With random mating, recall at multicopy regions dropped to 28.93% with read depth 3× and sample size 15, but remained high with a read depth of 5× at the same sample size (on average, recall was 78.4%) and with read depth 3× and a larger sample size of 50 (recall was on average 79.9%; supplementary fig. S3). Total recall (single-copy and multicopy SNPs combined) also remained high with inbreeding and with read depth 3× and sample size 15 (94.5% on average), and with random mating with either read depth 5× and sample size 15 or read depth 3× and sample size 50 (with a recall of 93.8% and 93.7%, respectively).

Plant material, short reads sequencing and variant calling

We collected cuttings and seeds from 85 individual plants in two Spanish populations of *Arabis alpina* from the Cantabrian Mountains, for a total of 36 individuals for population ES03 and 49 individuals for population ES04 (supplementary table S2). We rooted the cuttings in the greenhouse, and we extracted DNA from 100 mg of young leaves and meristems, following the DNeasy Plant Mini Kit protocol with minor adjustments. DNA integrity control, DNA library preparation and 150 bp paired end short reads nano ball sequencing was conducted at the Beijing Genomics Institute (BGI) for all samples. In particular, DNA concentration was quantified by fluorometer or microplate reader (e.g. qubit fluorometer, invitrogen). Sample integrity and purity were detected by agarose gel electrophoresis (concentration of agarose gel: 1%, voltage: 150 V, electrophoresis time: 40 minutes). 1 μg of genomic

DNA was randomly fragmented with covaris. The DNA fragments were selected by magnetic beads to an average size of 200-400 bp. Fragments were end repaired and then 3' adenylated. Adaptors were ligated to the ends of these 3' adenylated fragments. Fragments were amplified with PCR using the adaptors from the previous step. PCR products were purified by the magnetic beads. The double stranded PCR products were heat denatured and circularized by the splint oligo sequence. The single strand circle DNA (ssCir DNA) were formatted as the final library. The library was amplified with phi29 to make DNA nanoball (DNB). The DNBs were load into the patterned nanoarray and paired-end 150 base pairs reads were generated with combinatorial probe-anchor synthesis (cPAS).

SNP calling was performed using the GATK pipeline *v*4.2.0 [3]. Read groups were assigned, adapters were soft clipped, duplicates were marked and reads were aligned to the reference genome *Arabis alpina v5.1* [4] with bwa mem. Samples with genome-wide average read depth of 10 or less were excluded from the analysis. SNPs with read depth less than 5 and genotype quality less than 30 were set to missing values. Indels were removed and only biallelic sites were retained.

Identification of multicopy regions with ParaMask in *Oncorhynchus gorbuscha*, *Leptidea sinapis*, and *Oncorhynchus tshawytscha*

We downloaded publicly available *.vcf* files, which were not filtered based on excess of coverage or excess of heterozygousity and contained information on read depth (see Data Availability). The *L. sinapis* data set included only male individuals with two copies of Z-chromosomes, and singleton SNPs were already prefiltered. For both data sets we calculated average read depth per individual across all SNPs, including missing ones and excluded individuals with average read depth less than 10x. The genome-wide average read depth of the remaining individuals was 14.3x (range: 10.0x - 38.2x) and 14.1x (range: 10.2x - 21.8x) for *O. gorbuscha* and *L. sinapis*, respectively. For each individual, SNPs with read depth less than 5 or genotype quality less than 30 were set to missing values. In our analysis with ParaMask we excluded all SNPs with more than 10% missing genotypes and used default settings for all ParaMask scripts. For haplotype clustering we used chromosome sizes of the reference assembly of *O. gorbuscha* [5] and chromosome size specified in the linkage maps for *L. sinapis* [6]. For the Chinook Salmon VCF based on RAD-seq data, no additional filtering steps were applied. The

chromosome column was set equal to the tag ID, as RAD-seq tags were stored by ID. ParaMask was applied using a 30% missingness threshold to retain a comparable number of SNPs to those used in [7]. Since each SNP corresponded to a unique RAD tag, we omitted the clustering step of ParaMask. After filtering, we analyzed 19,306 SNPs, each located on distinct RAD tag sequences. Copy number annotations were available for 8,294 SNPs from [8]. In total, we identified 15.61% as multicopy SNPs, 51.74% as single-copy, and 32.64% remained unclassified.

Gene ontology enrichment analysis

We extracted the overlap between ParaMask calls and the genes annotated in the reference genome *Arabis alpina v5.1* [4]. We tested for enrichment of gene ontology (GO) categories for genes overlapping multicopy regions using the elim algorithm and Fisher's exact test implemented in the topGO package *v*2.42.0 [9]. Among the enriched GO categories, we found genes related to the integration of DNA segments into chromosomes (DNA integration) which can be related to transposon activity (including for instance a gene homologous to the retrovirus-related Pol polyprotein from transposon TNT 1-94 in *Vitis vinifera*, *POLX*). Further, we found an enrichment for defense response genes, including genes related to virus induced gene silencing and genes related to defense response to bacteria.

PacBio Hifi long-reads sequencing

To validate the calls of multicopy regions in *A. alpina* with ParaMask, we sequenced two of the 85 accessions, ES03-014 and ES04-014, with PacBio Hifi long-reads, one from each of the two populations, ES03 and ES04. For long read sequencing, genomic DNA was isolated from leaves with the NucleoBond HMW DNA kit (Macherey Nagel, Düren, Germany). A HiFi library was prepared according to the manual of the HiFi SMRTbell® Libraries using SMRTbell Express Template Prep Kit 2.0 (Pacific Biosciences, Menlo Park, CA, USA) with an initial DNA fragmentation using Diagenode Megaruptor® 3 (Denville, NJ, USA) and final library size binning into defined fractions by Blue Pippin with 0.75% agarose cassettes (Sage Science; Beverly, MA, USA). Size distribution was again controlled by Femto pulse system (Agilent, Santa Clara, CA, USA). Size-selected libraries were then sequenced at the Max Planck Genome-centre Cologne on a Sequel II device with Binding kit 2.0 and Sequel II Sequencing

Kit 2.0 for 30 h using two SMRT cells (Pacific Biosciences, Menlo Park, CA, USA).

Validation based on structural variant calling with long reads

To call structural variants, we aligned the HIFI long reads of individual genomes to the reference genome *Arabis alpina v5.1* [4] with minimap2 *v2.0.2* [10], using 20 threads and default settings. The aligned reads were then used as input for SV calling with sniffles2 *v2.2* [11] and cuteSV *v2.0.2* [12]. Both methods can detect duplications, insertions, deletions, inversions, and translocations. For both programs we set the minimum length of SVs to 35 bases, the default setting for sniffles2. After independently calling SVs with the two methods, we merged the resulting data set with SURVIVOR *v1.0.7* [13], which identifies SVs called by both cuteSV and sniffles2. We set the maximum allowed distance between breakpoints of the same SV in the two methods to 10% of the respective SV length.

To validate the calls from ParaMask, we generated high-confidence sets of multicopy genomic regions, and of single-copy regions, based on SV calling from long reads. For high-confidence multicopy regions, we focused on duplications, which are the SV class that always represents multicopy regions. To reduce the error rate associated with SV calling from long reads, we selected the duplications that were identified by both cuteSV and sniffles2. For comparison to ParaMask, we matched the minimum length of detected SVs (35 bp in SV calling), and we selected the ParaMask regions were either or both of the two genomes sequenced with long reads were heterozygote at SNPs inferred to be collapsed. To identify a set of high-confidence single-copy regions, we selected regions where neither cuteSV nor sniffles2 called any SV. To assess the agreement between ParaMask and high-confidence duplications identified through long-read SV calling, we calculated the proportion of SNPs located within each duplication that were classified as multicopy SNP. These percentages were then averaged across all SVs to obtain a normalized measure of agreement. In contrast to a genome-wide count of overlapping multicopy SNPs with duplications, this method is less biased by differences in SV length and it provides a more accurate estimate of how well ParaMask classifications align with long-read SVs. Additionally, the single-copy regions were filtered to a minimum length of 35 base pair to align with the multicopy regions and ensure higher confidence in the single-copy regions. ParaMask can detect multicopy regions in any of the 85 short reads genomes, while SVs were called from a subset of

two genomes sequenced with long reads. For an even comparison, we selected the subset of ParaMask calls were either or both of the two genomes sequenced with long reads were heterozygote at SNPs inferred to be collapsed. Finally, we downsampled the ParaMask calls to the multicopy regions longer than 35 base pair, to match the length threshold used in SV calling.

Long-reads genome assembly

Prior to genome assembly, we estimated heterozygosity and genome size using Jellyfish *v2.2.6* [14] and GenomeScope *v2.0* [15]. We assembled the two genomes sequenced with Pacbio Hifi *de novo* with Hifiasm *v0.19* [16] and Flye *2.9* [17] independently, with default parameters. To avoid the inclusion of haplotigs, i.e., sequences that represent alternative haplotypes, into the assembly, we purged the Flye contigs with PurgeDups *v1.2.5* [18]. The Hifiasm contigs were already purged by default during the assembly process. To increase the contiguity of the contigs, both assemblies were joined with quickmerge *v3.0* [19]. We set the parameter *-ml 5000* to filter contigs smaller than 5000 bp and we used the contigs from Flye as reference and those from Hifiasm as query. We scaffolded the contigs to the reference genome *A. alpina v5.1* [4] with RagTag *v2.1.0* [20] without chimeric contig correction and with parameter setting *-C* to merge all unassigned contigs to chromosome 0. We filled the gaps between pseudochromosomes with LR Gapcloser *v1.0* [21]. We evaluated the final assemblies with the Quality Assessment Tool (QUAST) *v5.0.2* [22] and assessed assembly completeness using Benchmarking Universal Single-Copy Orthologs (BUSCO) *v5.2.2* [23]. The long reads were mapped against the corresponding scaffolds using minimap2 *v2.0.2* [10], and read depth was calculated using samtools.

The assemblies were then screened for interspersed repeats and low complexity DNA using RepeatMasker *v4.0.9* [24], and the resulting repeats were lifted over to the reference genome using liftoff *v1.6.3* [25]. After aligning the raw long reads to their corresponding assemblies, we extracted the sequence of all regions exceeding 350x coverage, and a subset of the regions between 100x and 350x coverage, and blasted their sequences against the nt database.

Comparison between ParaMask and other available methods

To compare the performance of ParaMask with alternative methods, we used rCNV *v*.1.3.9 [7] and ngsParalog *v*1.3.4 [26, 27]. To analyse empirical and simulated data with rCNV, we applied the work flow and parameter settings as suggested on the GitHub page of the package and analysed each chromosome separately. We generated a normalized allele depth table using the 'median ratio' method. Based on this table, the allele information table was generated with the *allele.info* function. In order to detect SNPs in putative multicopy regions, we used the *dupGen* function under the expectation of an allele ratio of 0.5 and the default method for identifying excess heterozygosity. Finally, SNPs putatively located in multicopy regions were filtered using the K-means method integrated in the *cnv* function. The retained SNPs, classified as "cnv" were treated as multicopy SNPs.

In the analysis with ngsParalog on the empirical data of *A. alpina*, we generated a pileup file for the 85 accessions described above, using the mpileup function in samtools. In the analysis with ngsParalog on simulated data, we first generated a 1 Mbp reference genome that included the single-copy blocks interspersed with the first segment of each duplicated block using custom scripts. For each simulated sample, we generated a fasta file that included the single-copy blocks, and both segments of each duplicated block using either the reference or the derived sequence according to the allelic state from the simulations. With these fasta files we simulated Illumina sequencing reads in FASTQ format using ART Illumina *v*2.5.8 [28] (parameter setting: -ss HSXt -p -l 150 –sdev 10 –mflen 200). All FASTQ sequences were aligned to the simulated reference genome using bwa mem, and the resulting alignments were used to create a pileup file with SAMtools mpileup. For empirical and simulated data, we then used the ngsParalog calcLR function with parameters *-minQ 20 -minind 25 -mincov 1* to compute the log-likelihood ratio of mismapped reads for every SNP. Different chromosomes were run in parallel to shorten the run time. Based on the log-likelihood ratio per SNP, a chi-square test was performed under the null hypothesis that reads covering a particular site originate from a single-copy region. To classify multicopy SNPs with ngsParalog, we repeated the analysis with two different adjustments for multiple hypothesis testing and significance thresholds. First, we used the Benjamini-Hochberg correction with significance threshold of $p < 0.001$, as in [27]. Second, we used the Bonferroni correction with significance threshold of $p < 0.05$, as in [26].

After classifying SNPs into single-copy and multicopy with ngsParalog and rCNV, we compared these classifications to the results with ParaMask, and to structural variant (SV) calling with long reads. The comparison with SV calling was computed as described for the agreement between ParaMask and SV calling. For ngsParalog, because it relies on independent SNP calling, we computed the agreement with ParaMask on the SNPs that were identified in both pipelines. We further repeated all comparisons with ParaMask and with SV calling using the two different significance thresholds used in ngsParalog (described above). For rCNV, the SNPs used were initially the same as in ParaMask. However, rCNV not only classifies SNPs into single-copy and multicopy, but it also discards a subset of the SNPs as uncertain. To account for this, we first computed the comparison using all SNPs and distinguishing uncertain from classified SNPs. Second, we repeated the comparison after excluding all uncertain SNPs from both rCNV and ParaMask.

For comparisons on Chinook salmon RAD-seq data we focused on SNPs with annotated copy number status from [8] and we relied on the performance of HDplot and rCNV reported in the original manuscripts [1, 7]. Notably, some of the SNPs classified as multicopy by ParaMask and as single-copy in [8], had a read ratio of 0.25 and low heterozygote frequencies (supplementary fig. S12). Some of these SNPs may correspond to low-frequency multicopy regions that were not captured in the haploid families used to identify multicopy SNPs in [8].

Genomic summary statistics

We computed genomic summary statistics in simulated and empirical data, using custom scripts available at https://github.com/Fulgione-group/ParaMask_analysis. For θ_W, θ_π, and Tajima's D, we excluded sites with more than 10% missing genotypes, and we randomly subsampled 90% of the genotypes at each site, among the ones with non-missing calls. We scaled summary statistics to the number of non-missing sites, including invariant sites. For the allele frequency spectra (AFS and jAFS) we randomly subsampled sites to 100 chromosomes in simulated data, and to 30 chromosomes per population in empirical data, among the ones with non-missing calls. For empirical data, we polarized the spectra to the outgroup species *Arabis montbretiana*. We calculated the inbreeding coefficient F_{IS} as $1 - Ho/Hs$, where Ho is the observed heterozygosity, and Hs is within population diversity, using

hierfstat v0.5.11 [29] on simulated data, and using a custom script on empirical data. We calculated the weighted Weir and Cockerham F_{ST} estimator on empirical data using VCFtools v0.1.16 [30].

Demographic inference

To infer demographic histories, including split times between populations, migration rates, and effective population sizes, we used the allele frequency spectrum (AFS) based method dadi [31]. We generated joint AFS between the two Spanish populations of *A. alpina* after excluding genic regions (open reading frame and 2 kbp up- and downstream of all genes), because they are more likely to evolve under strong selection. To account for distortions in the AFS due to selfing, we used a single haplotype per diploid genome. We inferred demographic parameters with and without the multicopy regions identified with ParaMask, to test their effects on demographic inference. For both jAFS, we inferred parameters in 200 independent replicates with up to 50 iterations, for each of four demographic models of increasing complexity. We tested: 1) a model with a population split, no migration and constant population sizes, 2) a model with a population split, no migration and exponential changes in population sizes, 3) a model with a population split, no migration and a bottleneck, 4) a model with a population split, asymmetric migration and exponential changes in population size. We chose the best supported model for each jAFS on the basis of the replicate with the highest likelihood, according to the Akaike Information Criterion.

References

[1] McKinney GJ, Waples RK, Seeb LW, Seeb JE. Paralogs are revealed by proportion of heterozygotes and deviations in read ratios in genotyping-by-sequencing data from natural populations. Molecular Ecology Resources. 2017 Jul;17(4):656-69.

[2] Hartasánchez DA, Brasó-Vives M, Fuentes-Díaz J, Vallès-Codina O, Navarro A. SeDuS: segmental duplication simulator. Bioinformatics (Oxford, England). 2016 Jan;32(1):148-50.

[3] DePristo MA, Banks E, Poplin R, Garimella KV, Maguire JR, Hartl C, et al. A framework for

variation discovery and genotyping using next-generation DNA sequencing data. Nature Genetics. 2011 May;43(5):491-8.

[4] Jiao WB, Accinelli GG, Hartwig B, Kiefer C, Baker D, Severing E, et al. Improving and correcting the contiguity of long-read genome assemblies of three plant species using optical mapping and chromosome conformation capture data. Genome Research. 2017 May;27(5):778-86. Available from: http://genome.cshlp.org/lookup/doi/10.1101/gr.213652.116.

[5] Christensen KA, Rondeau EB, Sakhrani D, Biagi CA, Johnson H, Joshi J, et al. The pink salmon genome: uncovering the genomic consequences of a two-year life cycle. PLoS ONE. 2021;16(12 December):1-33.

[6] Torres AP, Höök L, Näsvall K, Shipilina D, Wiklund C, Vila R, et al. The fine-scale recombination rate variation and associations with genomic features in a butterfly. Genome Research. 2023;33(5):810-23.

[7] Karunarathne P, Zhou Q, Schliep K, Milesi P. A comprehensive framework for detecting copy number variants from single nucleotide polymorphism data: 'rCNV', a versatile r package for paralogue and CNV detection. Molecular Ecology Resources. 2023 Nov;23(8):1772-89.

[8] McKinney G, Seeb L, Larson W, Gomez-Uchida D, Limborg MT, Brieuc M, et al. An integrated linkage map reveals candidate genes underlying adaptive variation in Chinook salmon (*Oncorhynchus tshawytscha*). Molecular ecology resources. 2016;16(3):769-83.

[9] Alexa A, Rahnenfuhrer J. topGO: enrichment analysis for gene ontology; 2020. R package version 2.42.0.

[10] Li H. Minimap2: pairwise alignment for nucleotide sequences. Bioinformatics. 2018;34(18):3094-100.

[11] Smolka M, Paulin LF, Grochowski CM, Horner DW, Mahmoud M, Behera S, et al. Detection of mosaic and population-level structural variants with Sniffles2. Nature Biotechnology. 2024:1-10.

[12] Jiang T, Liu Y, Jiang Y, Li J, Gao Y, Cui Z, et al. Long-read-based human genomic structural variation detection with cuteSV. Genome biology. 2020;21(1):1-24.

[13] Jeffares DC, Jolly C, Hoti M, Speed D, Shaw L, Rallis C, et al. Transient structural variations have strong effects on quantitative traits and reproductive isolation in fission yeast. Nature communications. 2017;8(1):14061.

[14] Marçais G, Kingsford C. A fast, lock-free approach for efficient parallel counting of occurrences of k-mers. Bioinformatics. 2011;27(6):764-70.

[15] Ranallo-Benavidez TR, Jaron KS, Schatz MC. GenomeScope 2.0 and Smudgeplot for reference-free profiling of polyploid genomes. Nature communications. 2020;11(1):1432.

[16] Cheng H, Concepcion GT, Feng X, Zhang H, Li H. Haplotype-resolved de novo assembly using phased assembly graphs with hifiasm. Nature methods. 2021;18(2):170-5.

[17] Kolmogorov M, Yuan J, Lin Y, Pevzner PA. Assembly of long, error-prone reads using repeat graphs. Nature biotechnology. 2019;37(5):540-6.

[18] Guan D, McCarthy SA, Wood J, Howe K, Wang Y, Durbin R. Identifying and removing haplotypic duplication in primary genome assemblies. Bioinformatics. 2020;36(9):2896-8.

[19] Chakraborty M, Baldwin-Brown JG, Long AD, Emerson J. Contiguous and accurate de novo assembly of metazoan genomes with modest long read coverage. Nucleic acids research. 2016;44(19):e147-7.

[20] Alonge M, Lebeigle L, Kirsche M, Jenike K, Ou S, Aganezov S, et al. Automated assembly scaffolding using RagTag elevates a new tomato system for high-throughput genome editing. Genome biology. 2022;23(1):1-19.

[21] Marla SS, Mishra P, Maurya R, Singh M, Wankhede DP, Kumar A, et al. Refinement of draft genome assemblies of Pigeonpea (*Cajanus cajan*). Frontiers in Genetics. 2020;11:607432.

[22] Mikheenko A, Prjibelski A, Saveliev V, Antipov D, Gurevich A. Versatile genome assembly evaluation with QUAST-LG. Bioinformatics. 2018;34(13):i142-50.

[23] Simão FA, Waterhouse RM, Ioannidis P, Kriventseva EV, Zdobnov EM. BUSCO: assessing genome assembly and annotation completeness with single-copy orthologs. Bioinformatics. 2015;31(19):3210-2.

[24] Smit A, Hubley R, Green P. RepeatMasker Open-4.0 http://www. repeatmasker. org. RMDownload html. 2013.

[25] Shumate A, Salzberg SL. Liftoff: accurate mapping of gene annotations. Bioinformatics. 2021;37(12):1639-43.

[26] Linderoth T. In: Identifying population histories, adaptive genes, and genetic duplication from population-scale next generation sequencing. Doctoral dissertation, University of California, Berkeley; 2018. p. 5-39. Available from: https://escholarship.org/uc/item/5kp4q40k.

[27] Dallaire X, Bouchard R, Hénault P, Ulmo-Diaz G, Normandeau E, Mérot C, et al. Widespread deviant patterns of heterozygosity in whole-genome sequencing due to autopolyploidy, repeated elements, and duplication. Genome Biology and Evolution. 2023 Dec;15(12):evad229.

[28] Huang W, Li L, Myers JR, Marth GT. ART: a next-generation sequencing read simulator. Bioinformatics. 2012;28(4):593-4.

[29] Goudet J. hierfstat, a package for r to compute and test hierarchical F-statistics. Molecular Ecology Notes. 2005;5(1):184-6.

[30] Danecek P, Auton A, Abecasis G, Albers CA, Banks E, DePristo MA, et al. The variant call format and VCFtools. Bioinformatics (Oxford, England). 2011 Aug;27(15):2156-8.

[31] Gutenkunst RN, Hernandez RD, Williamson SH, Bustamante CD. Inferring the joint demographic history of multiple populations from multidimensional SNP frequency data. PLOS Genetics. 2009 Oct;5(10):e1000695. Publisher: Public Library of Science.
