# Supplementary material for: ParaMask: a new method to identify multicopy genomic regions, corrects major biases in whole-genome sequencing data
Source: Genome Biol. 2025 Oct 24;26:368. doi: 10.1186/s13059-025-03836-8 (PMC12551310; doi:10.1186/s13059-025-03836-8)
Supplement: Supplementary file 2 — Additional file 2: Supplementary Figures. This file includes Figures S1 to S14. [file 13059_2025_3836_MOESM2_ESM.pdf]

ParaMask: a new method to identify multicopy genomic regions, corrects major biases in whole-genome sequencing data

Additional file 2: Fig. S1-S14

Bastiaan Tjeng[1], Male Arimond[1], Helene Bråten Grindeland[1],

Andrea Dalla Libera[1], Andrea Fulgione[1,*]

October 6, 2025

[1]: Max Planck Institute for Plant Breeding Research, Carl-von-Linne-Weg 10, 50829, Cologne

***Corresponding author:** Andrea Fulgione, e-mail: fulgione@mpipz.mpg.de

Contacts:

Bastiaan Tjeng: btjeng@mpipz.mpg.de

Male Arimond: marimond@mpipz.mpg.de

Helene Bråten Grindeland: hgrindeland@mpipz.mpg.de

Andrea Dalla Libera: adallalibera@mpipz.mpg.de

Andrea Fulgione: fulgione@mpipz.mpg.de

Fig. S1

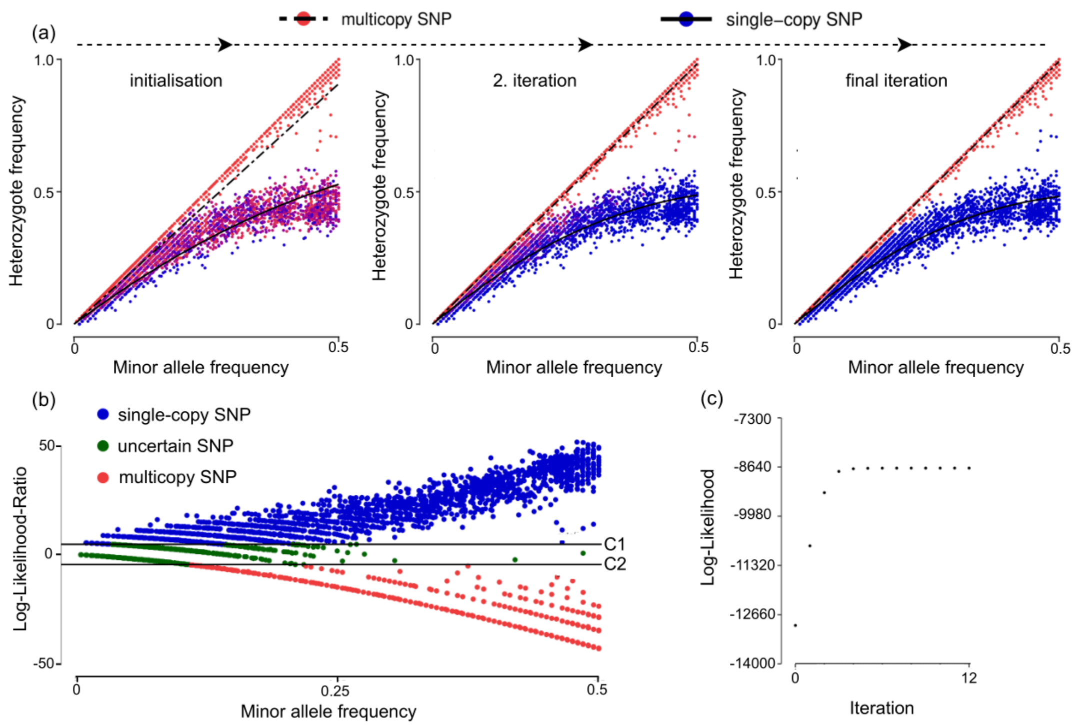

Schematic of an EM run on simulated genomes with 10% duplications and random mating. **a:** Heterozygote frequency as a function of the minor allele frequency, colored in a gradient scale proportional to the posterior distribution of the latent factor (weights) at the final EM iteration. The color scale varies from red (SNPs likely to belong to multicopy regions) to blue (SNPs likely to belong to single-copy regions). Curves represent the model fit for the relationship between heterozygote and allele frequencies at single-copy (solid line) and multicopy (dashed line) SNPs. **b:** Log likelihood-ratio (LLR) of belonging to single-copy region. Classification based on LLR cutoffs (C1: upper cuttoff, C2: lower cutoff). SNPs classified as single-copy are in blue, SNPs classified as multicopy are in red and uncertain SNPs are in green. **c:** Convergence of the log Likelihood through multiple EM cycles.

Fig. S2

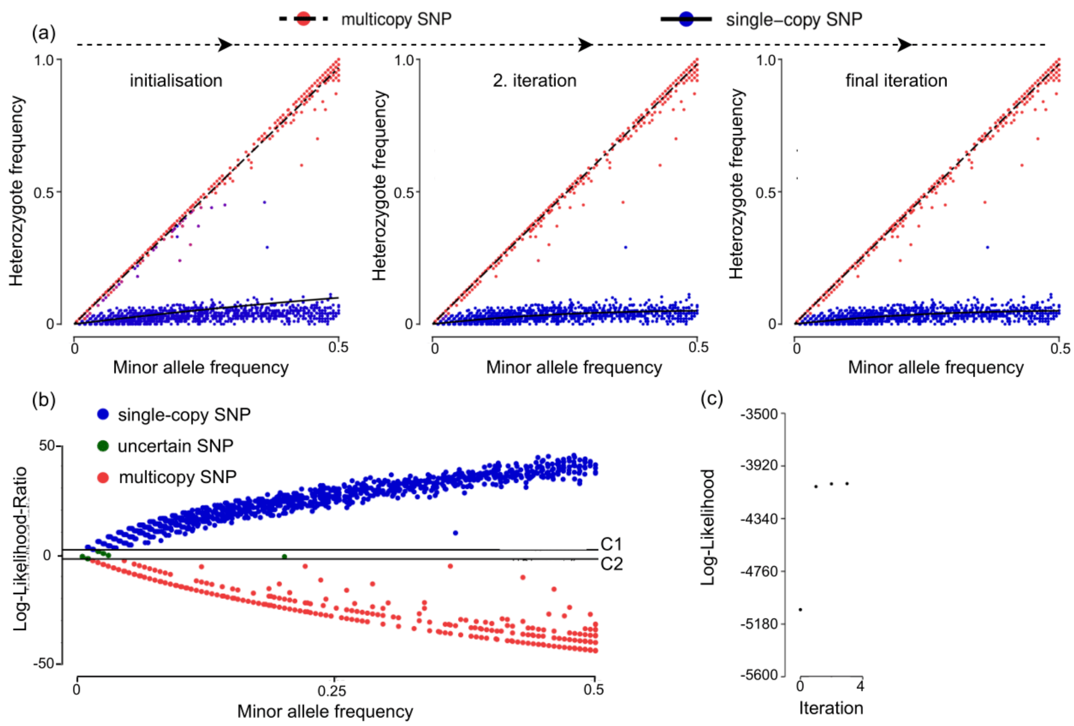

Schematic of an EM run on simulated genomes with 10% duplications and inbreeding ($F_{IS} = 0.9$). **a:** Heterozygote frequency as a function of the minor allele frequency, colored in a gradient scale proportional to the posterior distribution of the latent factor (weights) at the final EM iteration. The color scale varies from red (SNPs likely to belong to multicopy regions) to blue (SNPs likely to belong to single-copy regions). Curves represent the model fit for the relationship between heterozygote and allele frequencies at single-copy (solid line) and multicopy (dashed line) SNPs. **b:** Log likelihood-ratio (LLR) of belonging to single-copy region. Classification based on LLR cutoffs (C1: upper cuttoff, C2: lower cutoff). SNPs classified as single-copy are in blue, SNPs classified as multicopy are in red and uncertain SNPs are in green. **c:** Convergence of the log Likelihood through multiple EM cycles.

Fig. S3

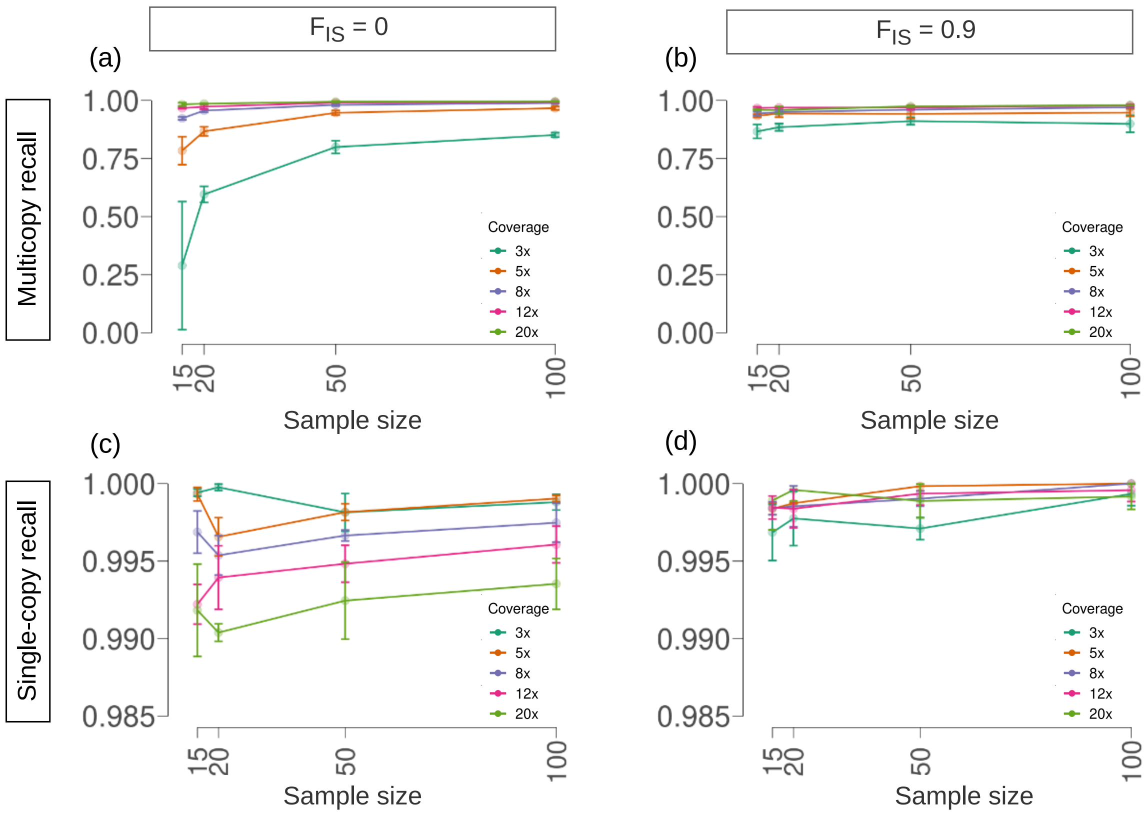

Average recall of ParaMask for single-copy, multicopy and all SNPs, with different sample sizes and different sequencing depth. **a**: Recall for multicopy SNPs with random mating ($F_{IS} = 0$). **b**: Recall for multicopy SNPs with inbreeding ($F_{IS} = 0.9$). **c**: Recall for single-copy SNPs with random mating. **d**: Recall for single-copy SNPs with inbreeding. Colors indicate different sequencing depth (Coverage). Error bars show standard deviation across three replicates. Note: The EM step failed to converge for simulations with 10 samples and random mating and produced no results.

Fig. S4

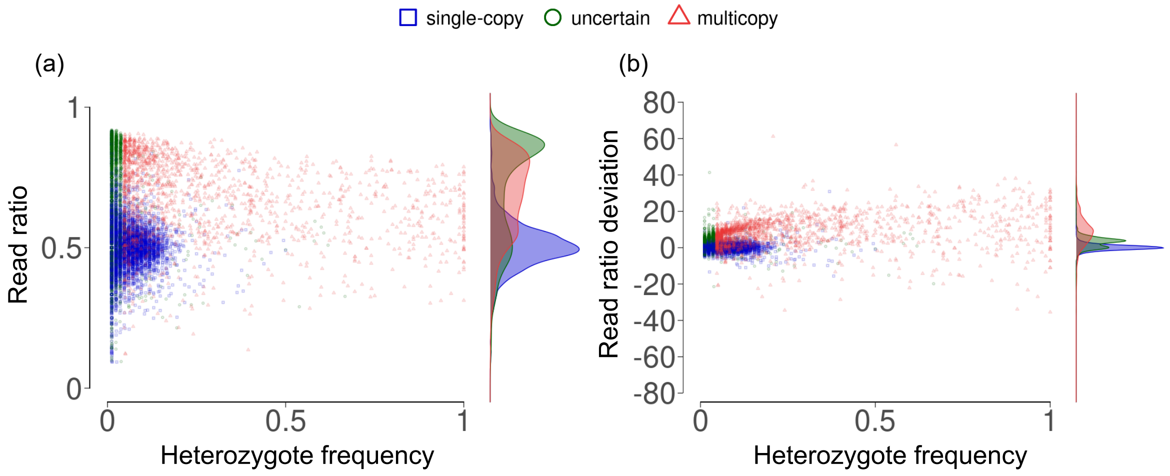

Read ratios and read-ratio deviations of heterozygotes as a function of heterozygote frequency for 10k randomly chosen SNPs from two *A. alpina* populations. Colors represent the classification by the EM algorithm: single-copy SNPs are in blue, multicopy SNPs are in red and uncertain SNPs are in green. **a:** Read ratios and marginal frequencies of read ratios. **b:** read-ratio deviations (D), calculated by the z-score and marginal frequencies of D.

Fig. S5

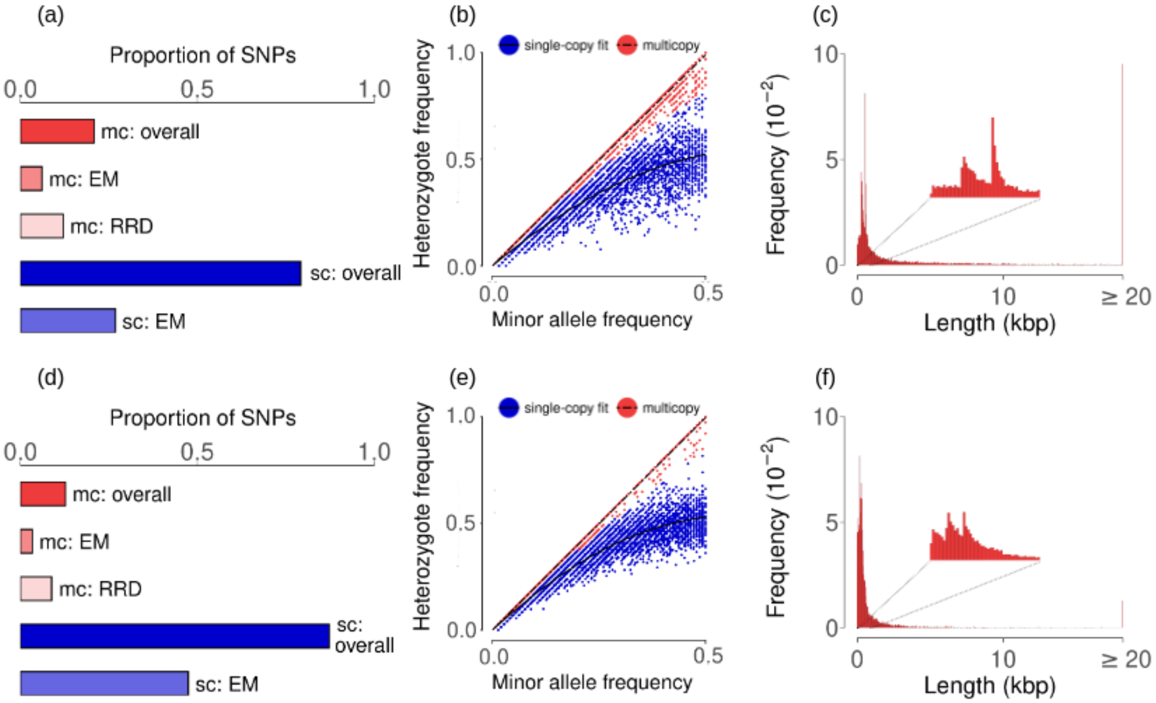

Identification of single-copy and multicopy regions in *O. gorbuscha* (top row, a-c) and *L. sinapis* (bottom row, d-f) populations with ParaMask. **a,d:** Proportion of SNPs identified as multicopy (mc) or single-copy (sc). The bars represent proportions for the overall ParaMask procedure (dark colors), after only the first EM step (mid-to-light colors) and after testing for read-ratio deviations (only for multicopy SNPs, light color). **b,e:** Heterozygote frequency as a function of the minor allele frequency, colored in a gradient scale proportional to the posterior distribution of the latent factor (weights) at the final EM iteration. The color scale varies from red (SNPs likely to belong to multicopy regions) to blue (SNPs likely to belong to single-copy regions). Curves represent the model fit for the relationship between heterozygote and allele frequencies at single-copy (solid line) and multicopy (dashed line) SNPs. **c,f:** Histogram of the length distribution of multicopy haplotypes for a binsize 40 bp. Inlets show the histogram for haplotype length up to 1 kbp for a binsize of 20 bp.

Fig. S6

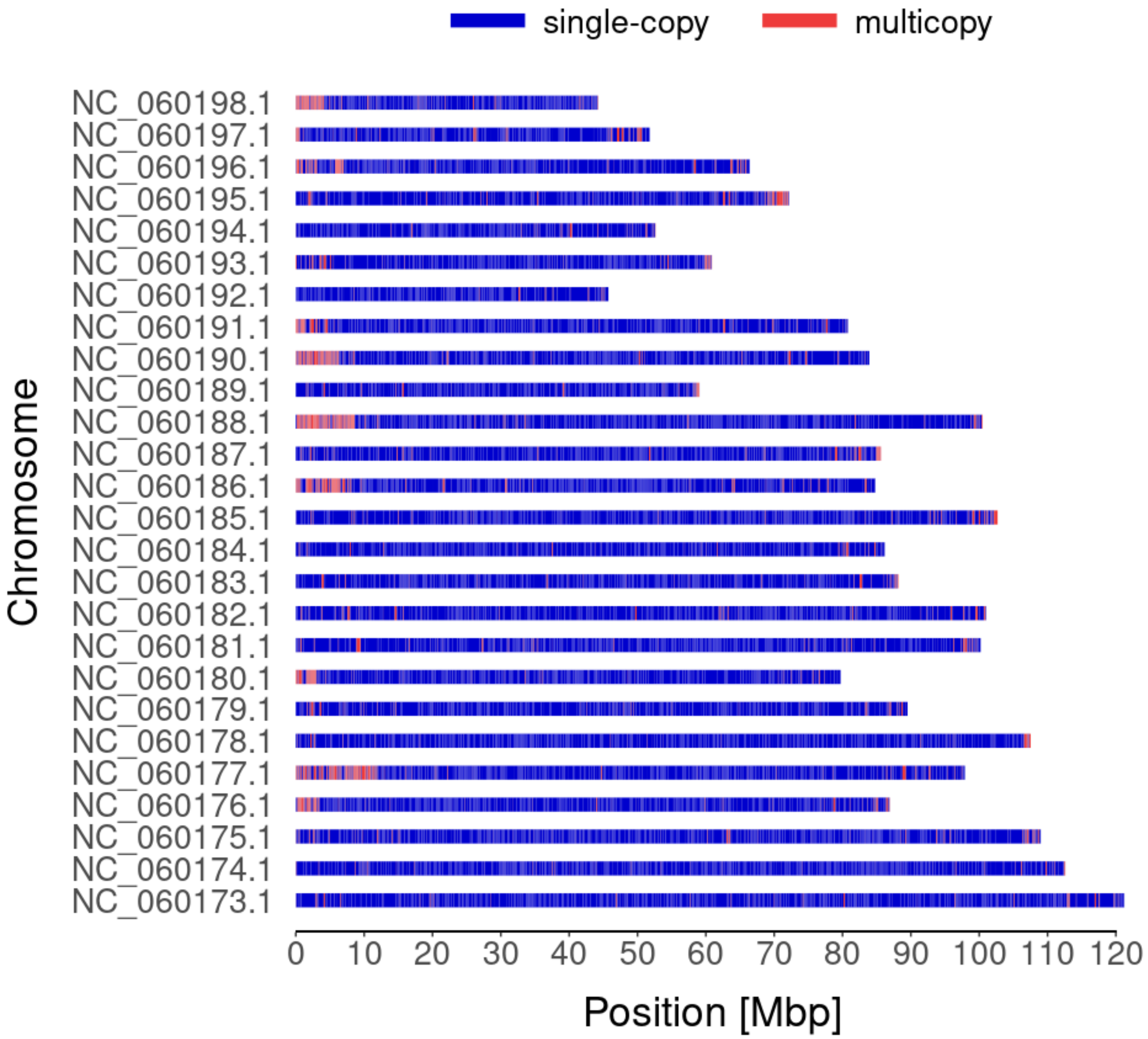

Distribution of multicopy regions in *O. gorbuscha* identified by ParaMask across autosomal chromosomes. Single-copy regions are colored in blue and multicopy regions are colored in red.

Fig. S7

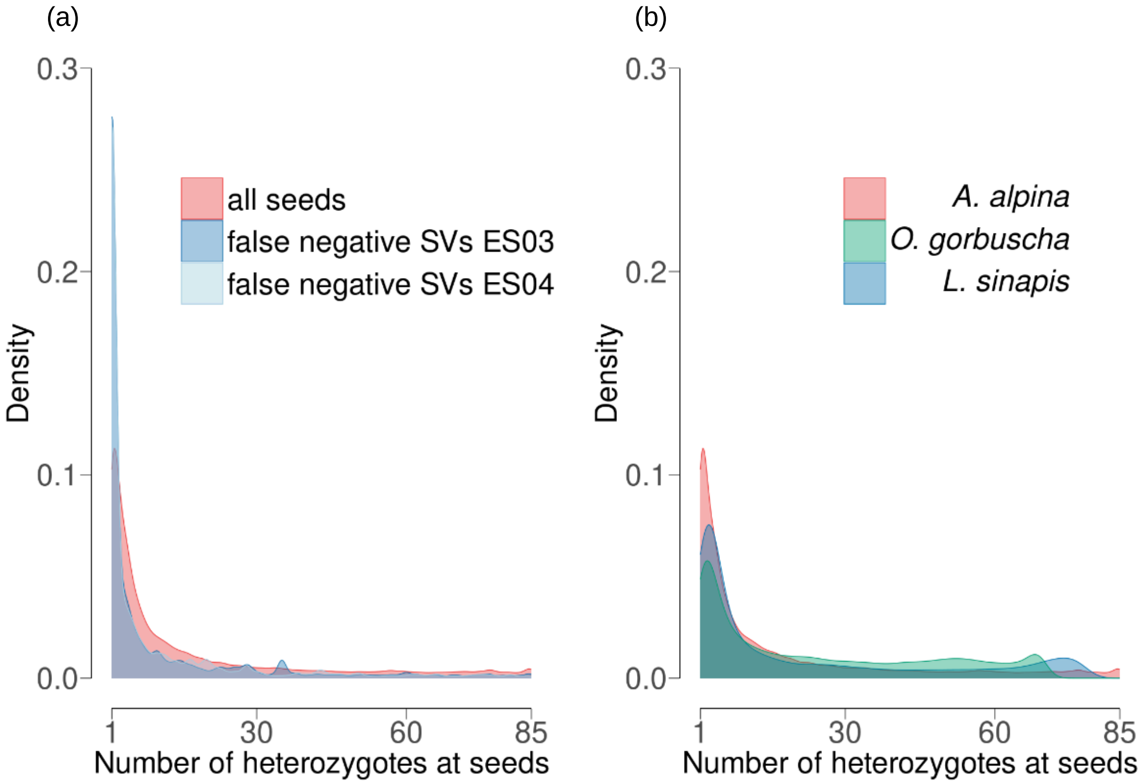

Density of the number of heterozygote individuals at SNPs classified as multicopy by the EM or after testing for read-ratio deviations (seeds). **a:** Density of heterozygotes for all seeds (red), and for seeds adjacent to single-copy SNPs within duplicated regions identified with SV calling from long reads for samples ES03-014 and ES04-014 (blue shades). **b:** Density of heterozygotes at SNPs classified as multicopy for *A. alpina* (red), *O. gorbuscha* (green), and *L. sinapis* (blue).

Fig. S8

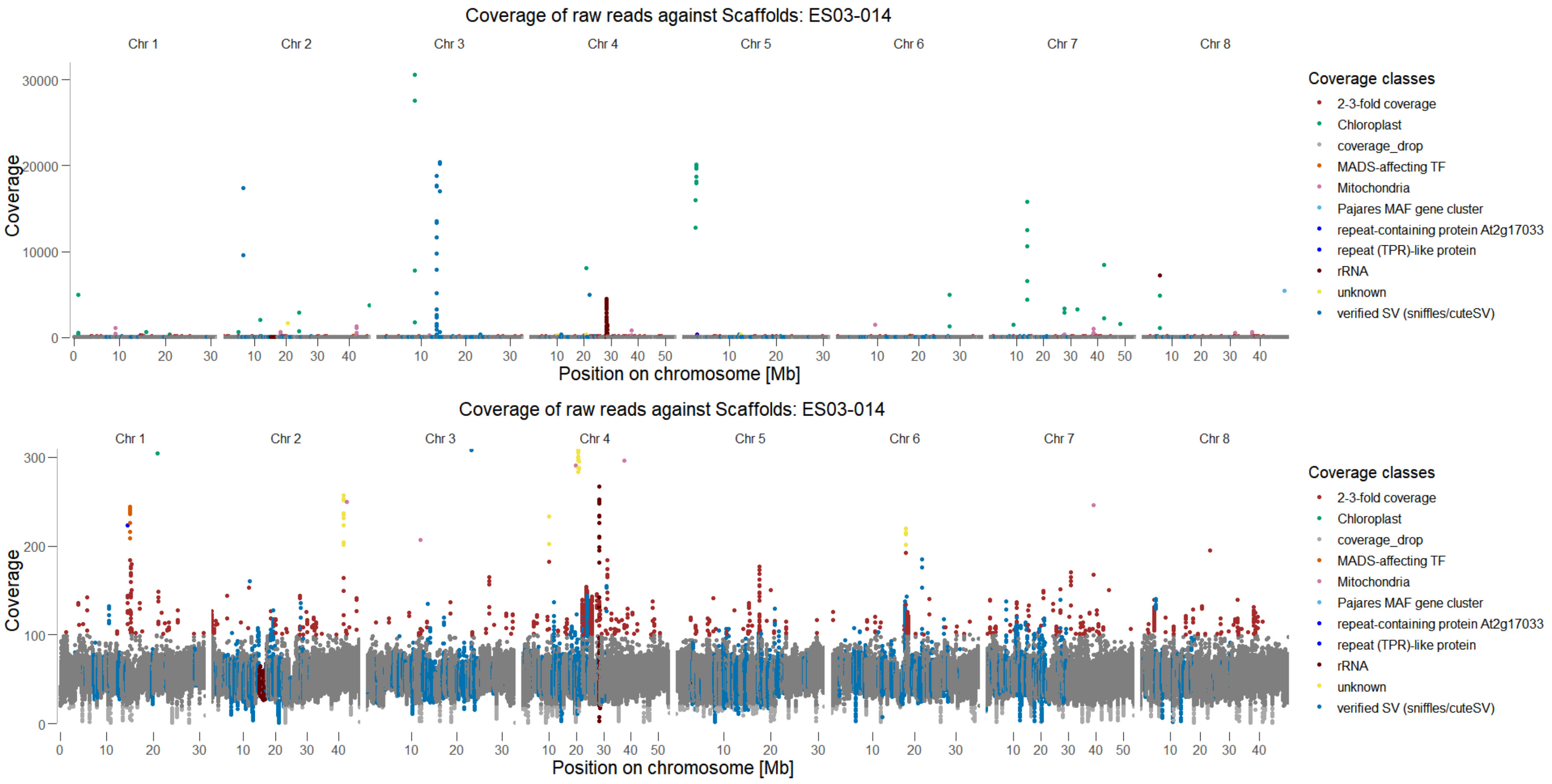

Coverage of long reads aligned to the assembly generated on the same data set for accession ES03-14. Peaks of increased coverage represent collapsed multicopy regions in long-reads assemblies. The legend shows the composition of some of the peaks based on blast hits. The bottom panel shows the subset of the top panel with coverage between 0 and 300, to increase resolution in this range.

Fig. S9

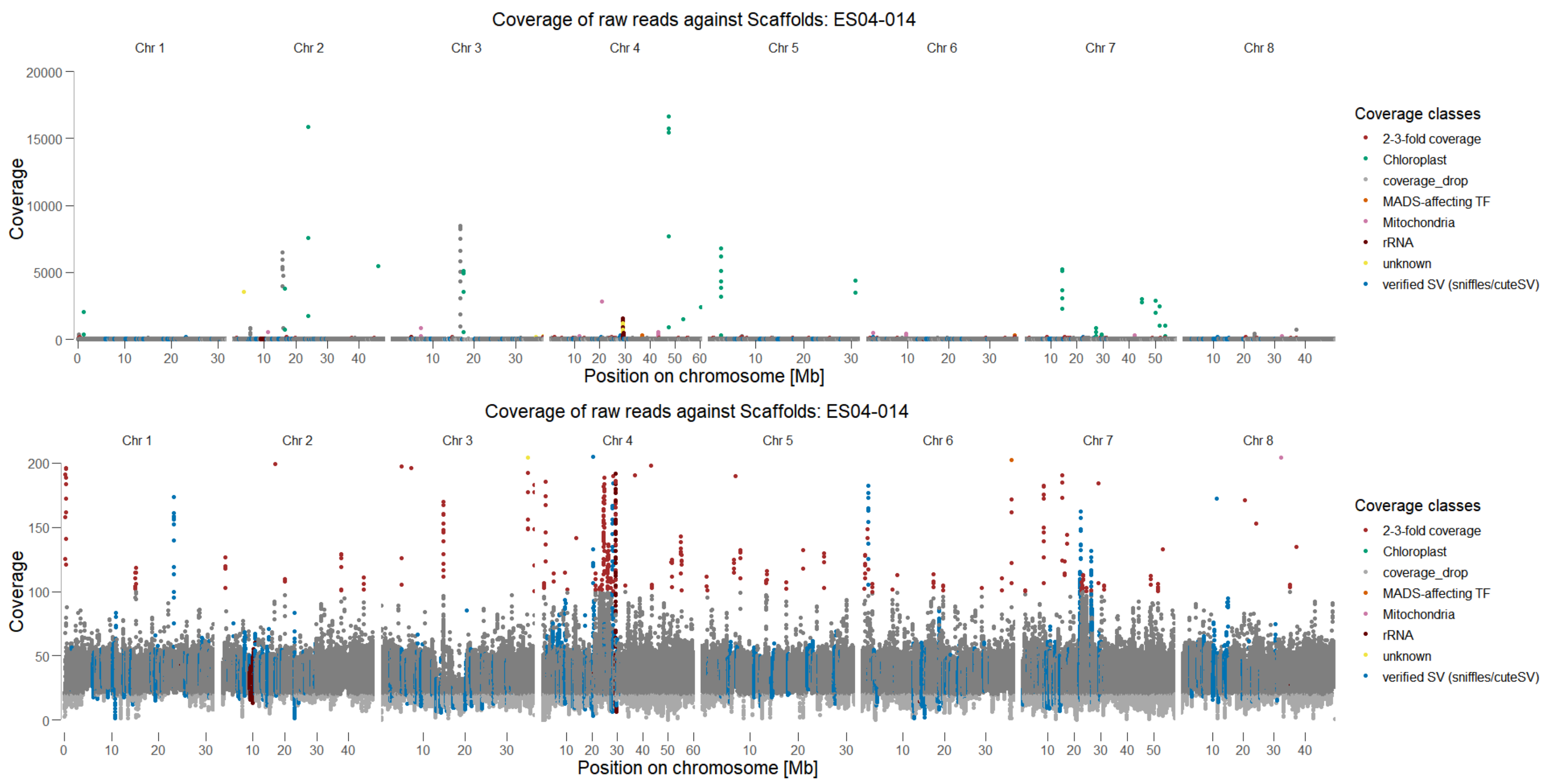

Coverage of long reads aligned to the assembly generated on the same data set for accession ES04-14. Peaks of increased coverage represent collapsed multicopy regions in long-reads assemblies. The legend shows the composition of some of the peaks based on blast hits. The bottom panel shows the subset of the top panel with coverage between 0 and 300, to increase resolution in this range.

Fig. S10

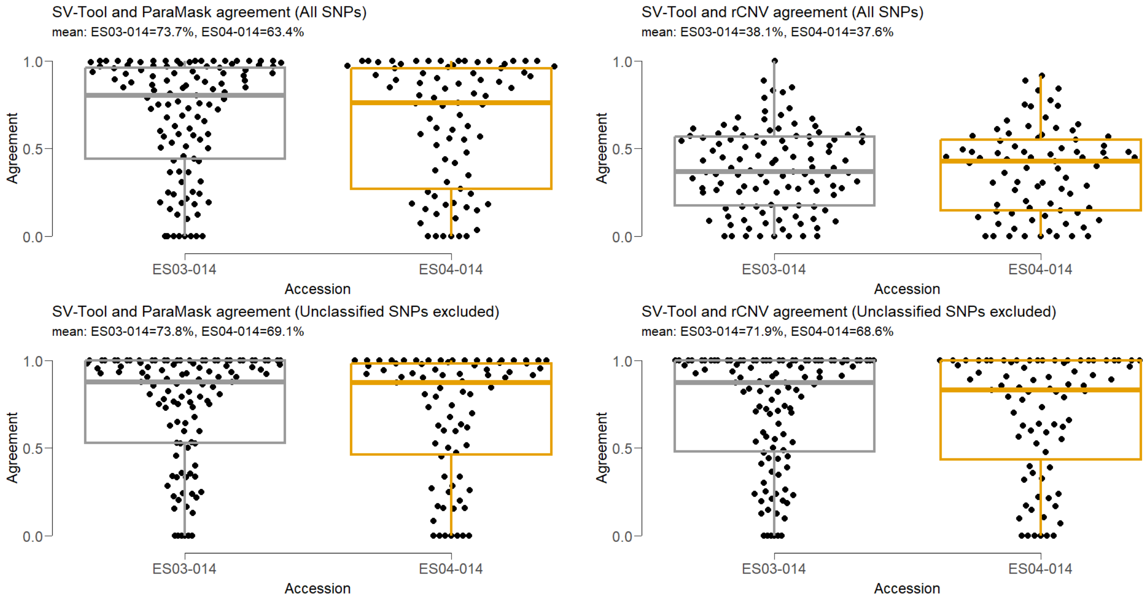

Comparison between rCNV or ParaMask calls and structural variants (SV) calling from long reads in two spanish accessions (ES03-14, ES04-14). For each accession, the proportion of all SNPs within duplications that are correctly classified by ParaMask (top-left) and by rCNV (top-right) are shown. The same analysis is shown at the bottom of the figure, after excluding SNPs classified as uncertain by rCNV, for ParaMask (bottom-left) and rCNV (bottom-right). Single points represent single SVs, and boxplots show median, 25th and 75th quantiles.

Fig. S11

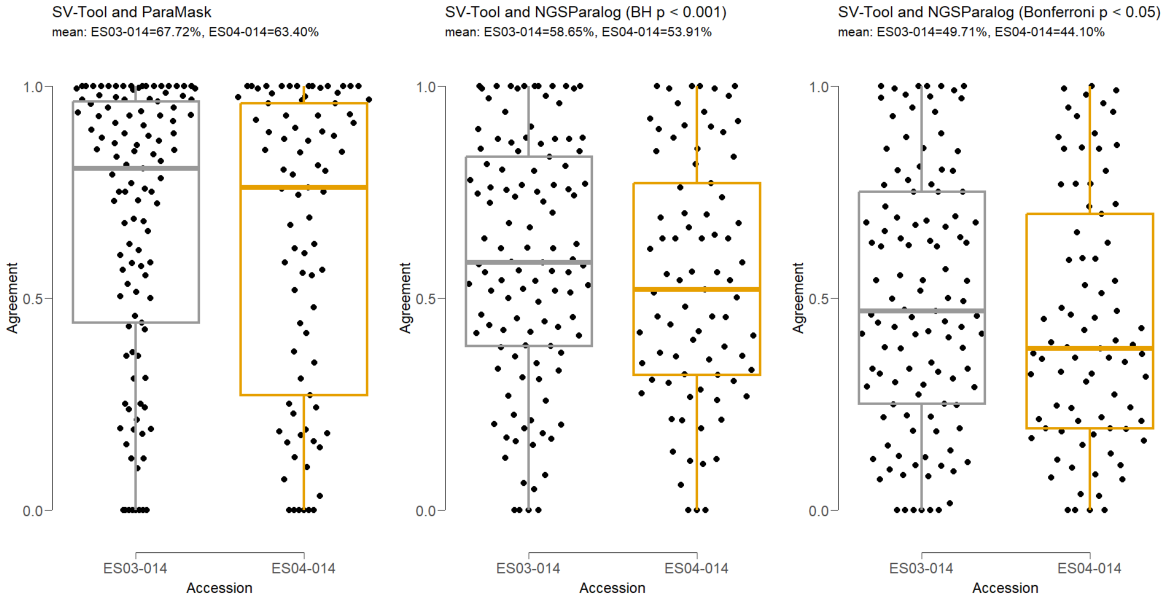

Comparison between ngsParalog or ParaMask calls and structural variants (SV) calling from long reads in two spanish accessions (ES03-14, ES04-14). For each accession, the proportion of SNPs within duplications that are correctly classified by ParaMask (left), by ngsParalog using Benjamini-Hochberg (BH) adjustment and significant level of $p < 0.001$ (middle), and by ngsParalog using bonferroni adjustment and significant level of $p < 0.05$ (right) are shown. Single points represent single SVs, and boxplots show median, 25th and 75th quantiles.

Fig. S12

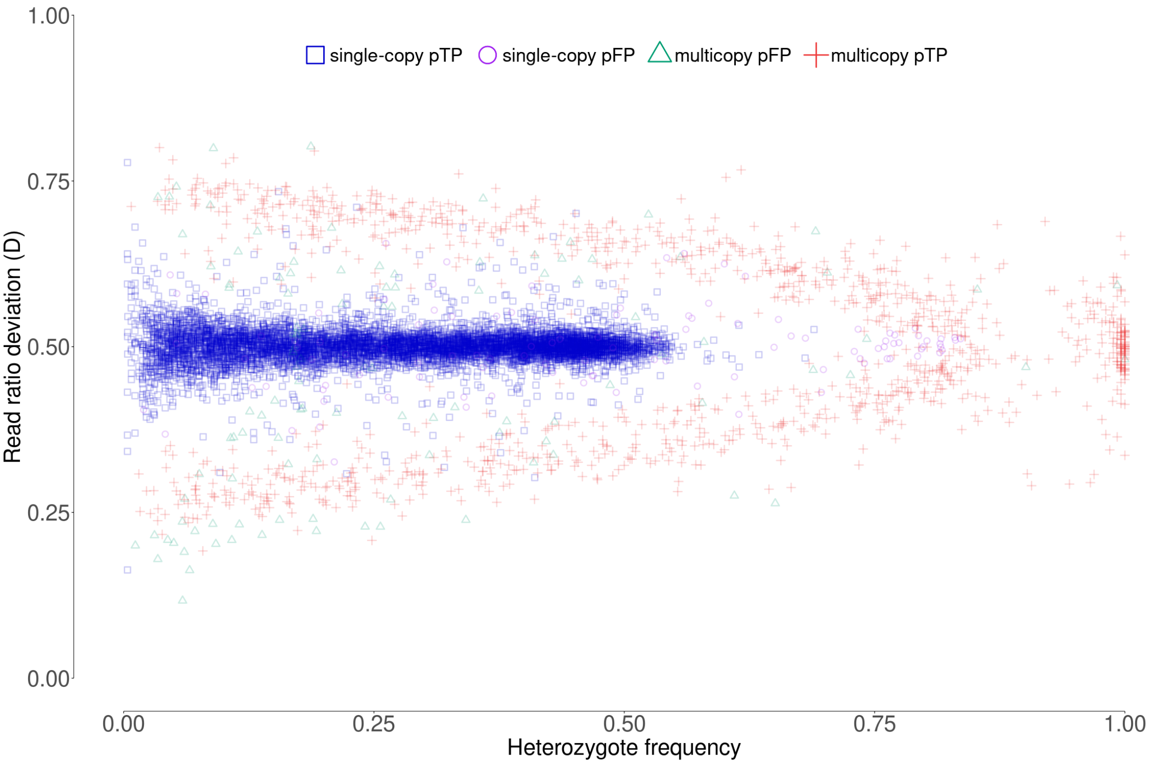

Classification of Chinook salmon RAD-seq SNPs using ParaMask without the clustering step. Heterozygote frequency and read-ratio deviation are plotted for every SNP. Pseudo true and false positives are inferred by comparison with known multicopy SNPs from [**?**]. Pseudo true positive single-copy SNPs are shown as blue rectangles, pseudo false positive single-copy SNPs as purple circles, pseudo false positive multicopy SNPs as green triangles, and pseudo true positive multicopy SNPs as red crosses.

Fig. S13

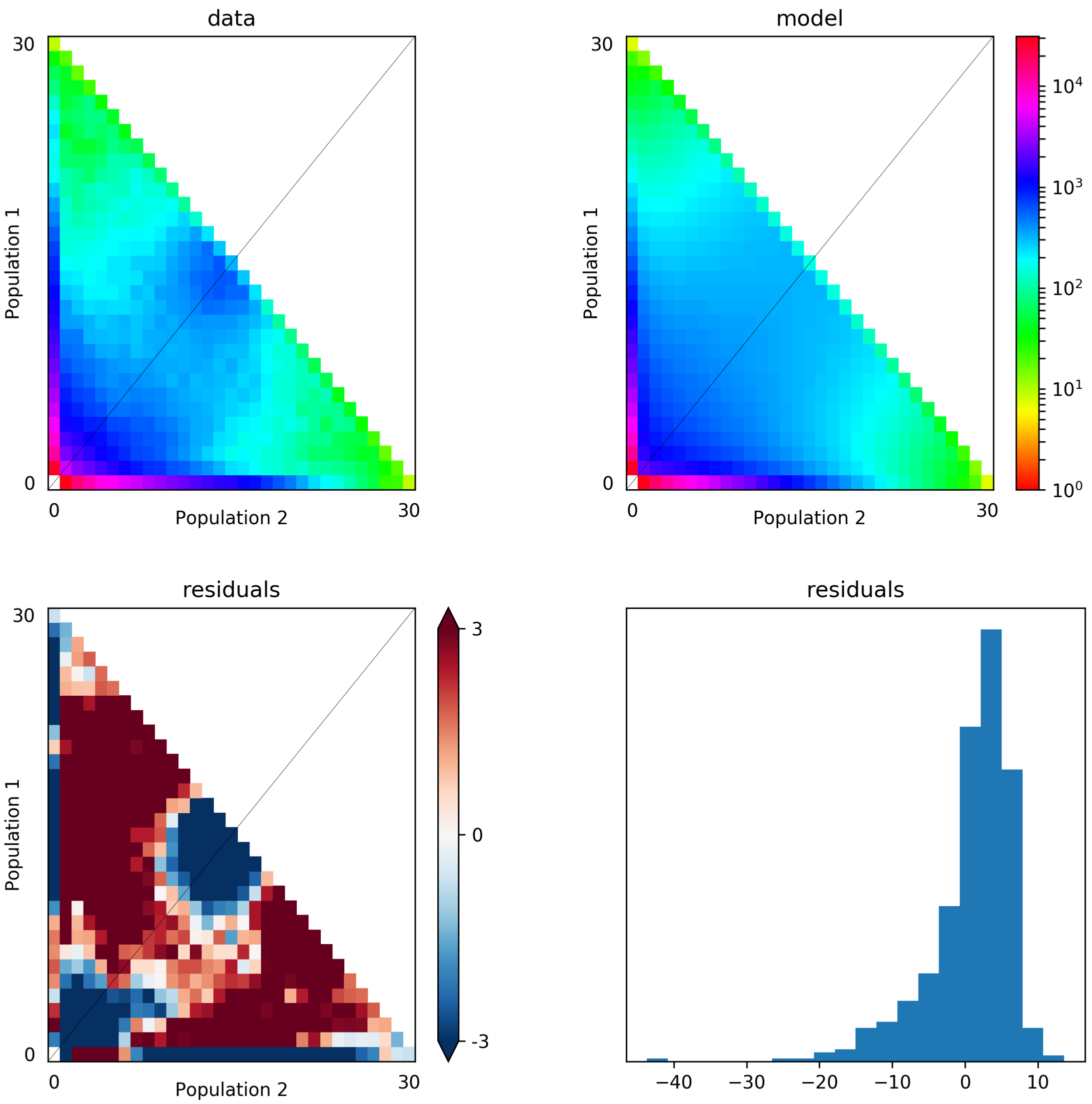

Inference of the demographic history of spanish *A. alpina* populations with dadi and with no filtering for multicopy regions. Data: observed folded joint allele frequency spectrum (jAFS); model: predicted jAFS of the optimised model; residuals: difference between predicted and observed SNPs across allele frequency categories.

Fig. S14

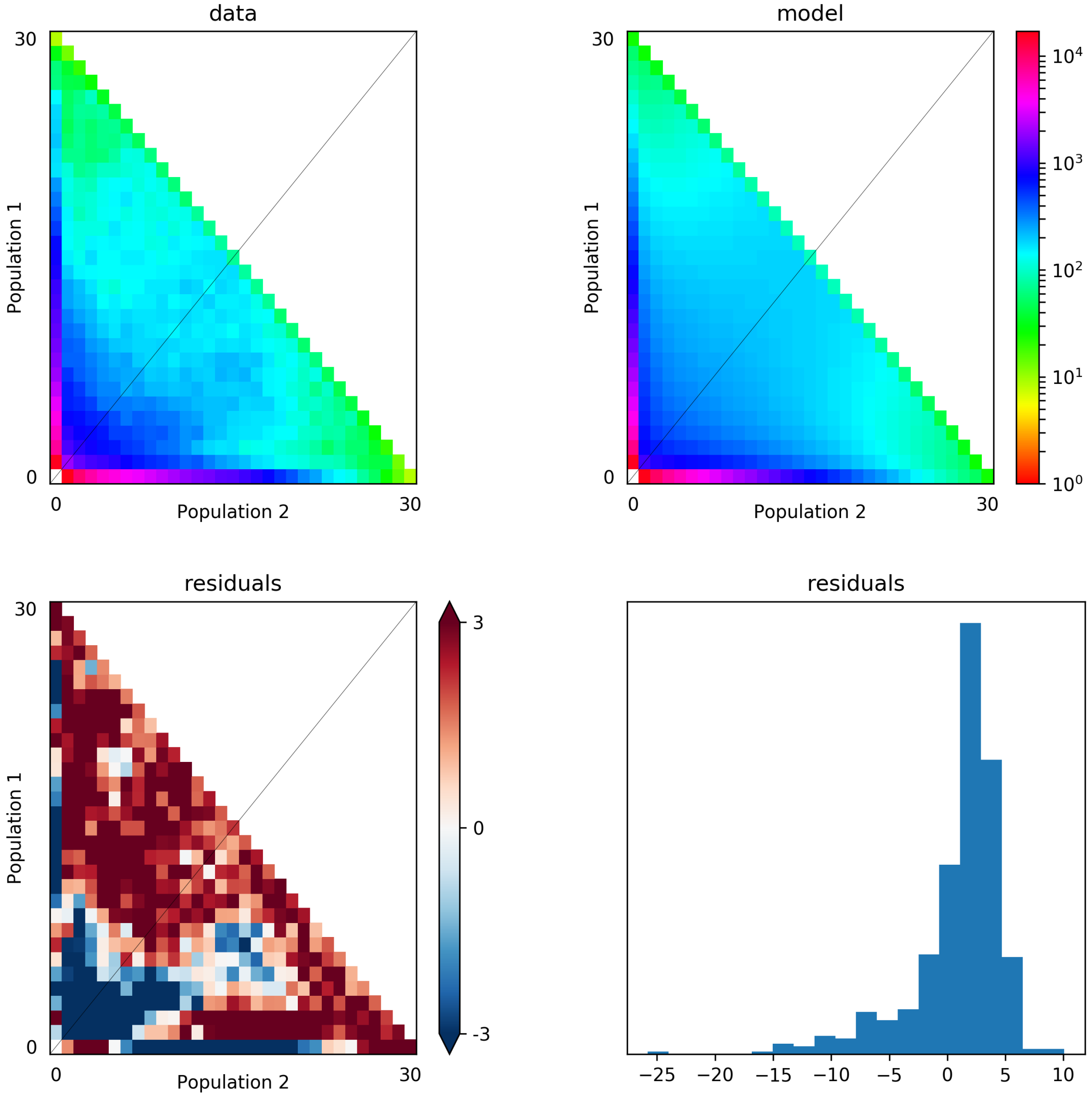

Inference of the demographic history of spanish *A. alpina* populations with dadi, after filtering for multicopy regions with ParaMask. Data: observed folded joint allele frequency spectrum (jAFS); model: predicted jAFS of the optimised model; residuals: difference between predicted and observed SNPs across allele frequency categories.
